# Supplementary material for: Groundwater level observations in 250,000 coastal US wells reveal scope of potential seawater intrusion
Source: Nat Commun. 2020 Jun 26;11:3229. doi: 10.1038/s41467-020-17038-2 (PMC7319989; doi:10.1038/s41467-020-17038-2)
Supplement: Supplementary file 1 — Supplementary Information [file 41467_2020_17038_MOESM1_ESM.pdf]

Supplementary Information for: “Groundwater level observations in 250,000 coastal US wells reveal scope of potential seawater intrusion” by Jasechko et al.

This supplementary information presents, in order (A) Supplementary Notes, (B) Supplementary Figures, (C) Supplementary Tables.

## (A) Supplementary Notes

These Supplementary Notes (depicted in Supplementary Figure 1) describe (i) well water level data sources and where to access these data (Supplementary Note 1), (ii) how we assured that accurate land surface elevations were captured by the digital elevation model used to determine wellhead elevations (Supplementary Note 2), (iii) how we tested for the quality of well water level measurements made in constructed wells (Supplementary Note 3), (iv) sensitivity of our results to the selected distance from a well to the coast, as used in Figure 4 (main text; Supplementary Note 4), (v) the relative spatial coverage of monitoring well data versus driller report water level data (Supplementary Note 5), (vi) the influence of imperfect well locations on the results we present in the main text (Supplementary Note 6), (vii) compilation of coastal hydrogeologic investigations (Supplementary Note 7), (viii) comparison of our results against the compilation local-scale studied (Supplementary Note 8), and (ix) observed changes in monitoring well water levels over time (Supplementary Note 9).

**Note 1: Well water level data synthesis and where data can be accessed**
Description of groundwater well water level database availability and accessibility

## Note 1. Well water level data synthesis and where data can be accessed

Groundwater well water level data were downloaded from national-, state- and county-level sources. This Supplementary Note 1 presents an overview of each dataset and, specifically, information about the agencies hosting each database so that others may gain access to the data analyzed here through contact with each agency.  We describe quality-control steps applied to each database. These steps were applied to the entire database; therefore, a single record may be excluded in one more than one step (i.e., a record may be excluded on the basis of poor latitude-longitude information, and also on the basis of inadequate well completion date information). Although our study focuses only on coastal states, we compiled well water level data for all states (including those inland) in order to develop the largest sample size possible for our comparison of well water levels reported in well completion report versus those measured in monitoring wells (i.e., a larger sample size for our assessment of the quality of driller report water levels presented in Note 3).

Some of the data use agreements linked to the state-level groundwater well databases do not allow us to reproduce these databases for download; here we detail how the primary well water level data can be downloaded either via (i) direct download from web pages, or (ii) personal communication with state or sub-state agencies. Each subsection details how the data were accessed and prepared for analyses (see also information about dataset sources detailed in Perrone, D., & Jasechko, S. Deeper well drilling an unsustainable stopgap to groundwater depletion. *Nature Sustainability* **2,** 773-782 (2019)). At the end of each subsection, we include a short statement providing directions and hyperlinks to database access points or contact information for database managers. For information about data quality control for several western states see Perrone, D., & Jasechko, S. (2017). Dry groundwater wells in the western United States. Environmental Research Letters, 12(10), 104002.

### Alabama

We downloaded n=1,355 records well completion records for Alabama December 4, 2017 via personal communication with a representative from the Geological Survey of Alabama. We acknowledge and thank the Geological Survey of Alabama for sharing these data. We did not identify replicate records for removal in the dataset, as a clear approach to doing so was not apparent. We did not exclude any records from the Alabama state groundwater well completion dataset on the basis of incomplete or implausible well location data; all recorded well locations lie within state boundaries.

For direct contact information with database managers see: www.gsa.state.al.us/inter/staff

### Alaska

Alaska groundwater well water level records were downloaded via personal communication with the Alaska Department of Natural Resources. We acknowledge Alaska's Department of Natural Resources for making these data available. No records were excluded based on potential replication. Most latitude and longitude fields were blank in the database; to geolocate the wells, we analyzed their township-range-section data, where available (https://sdms.ak.blm.gov/sdms/data_protracted_grid_gis.html). We compared our geolocated latitude/longitude data (based on township-range-section information) against recorded latitudes and longitudes and found nearly all (95%) calculated coordinates were within 3 km of recorded latitude and longitudes, among the small sample of records with recorded latitudes and longitudes. Therefore, we infer that most well locations for Alaska are uncertain to ~1-3 km. We did not analyze the Alaskan records extensively in this analysis.

Primary contact for information about well completion data: Kevin Petrone (see http://dnr.alaska.gov/mlw/water/hydro/components/regional-offices.cfm)

## California

We applied the methodology and downloaded data from the same sources described by Perrone and Jasechko (Perrone, D. & Jasechko, S. Dry groundwater wells in the western United States. *Environmental Research Letters* **12,** 104002 (2017); data available: https://water.ca.gov/Programs/Groundwater-Management/Wells). We removed all records lacking latitude or longitude data, or with latitude-longitude data corresponding to locations outside of California.

Data available for direct download from https://water.ca.gov/Programs/Groundwater-Management/Wells

## Delaware

Delaware well completion records were obtained through personal communication with the Delaware Geological Survey. We acknowledge the Delaware Geological Survey's support. Data were downloaded November 29, 2017 from https://data.delaware.gov/Energy-and-Environment/Well-Permits/2655-qn8j. We excluded 10,896 well construction records without latitude and longitude (or UTM) data; we also excluded 129 records with a recorded location falling outside of the boundaries of Delaware.

Data available for direct download from https://data.delaware.gov/Energy-and-Environment/Well-Permits/2655-qn8j

## Florida (1/5): Northwest Florida Water Management District

We downloaded Northwest Florida Water Management District well water level data on November 29, 2017 from https://www.nwfwater.com/Permits/Well-Permits/Setbacks-Fees-Maps/Well-Data-from-Submitted-Completion-Reports. We excluded 54,820 records with missing location data or because the locations were outside of state boundaries.

Data available for direct download from https://www.nwfwater.com/Permits/Well-Permits/Setbacks-Fees-Maps/Well-Data-from-Submitted-Completion-Reports

## Florida (2/5): Southwest Florida Water Management District

We downloaded data November 29, 2017 from the Southwest Florida Water Management District Open Data Portal: https://data-swfwmd.opendata.arcgis.com/datasets/31daef8c0a8a4352bb5b37abd9c39527_7. We excluded 2 records with recorded latitude-longitude pairings leading to a location over the ocean.

Data available for direct download from https://data-swfwmd.opendata.arcgis.com/datasets/well-construction-permits

## Florida (3/5): Suwannee River Water Management District

We downloaded well completion data via personal communications with the Suwannee River Water Management District December 1, 2017 (http://www.mysuwanneeriver.com/). We excluded 79 records with recorded locations outside of the state of Florida.

Data available via personal communication with database manager; for contact information see http://www.mysuwanneeriver.com/Directory.aspx?did=30

## Florida (4/5): St. Johns River Water Management District

We downloaded state-level well completion data December 4, 2017 via personal communications with the St. Johns River Water Management District (https://www.sjrwmd.com). We excluded 10,917 records because these records had either (i) blank values listed under the location heading, or (ii) because the recorded locations lay outside of state boundaries. We also excluded 96 records with latitude and

longitude data that appeared to be recorded only to the nearest integer (e.g., we excluded a record with a reported location of exactly 81.00000 degrees W).

Data provided via personal communication with the St. Johns River Water Management District; database manager is Wesley A. Curtis, P.G..

## Florida (5/5): South Florida Water Management District

No single database exists for the South Florida Water Management District. Instead, we contacted individuals at each of the 17 counties within the South Florida Water Management District; we received data from 12 of these 17 counties. Many of the compiled county-level datasets are biased strongly toward wells constructed in recent years; further, the extensiveness of record-keeping varies widely across county boundaries, meaning that the quality of data available for the South Florida Water Management District differs from neighboring Water Management Districts. In all, we analyzed just over 100,000 well records for the South Florida Water Management District. Notably, the comprehensiveness of each county's record keeping process varied widely, meaning some counties reported tens of thousands of records collected over decades, and others provided only a few hundred records (e.g., Orange County, where data are only available for wells constructed after the year 2016). Primary points of contact for the South Florida Water Management District may be found at: https://www.sfwmd.gov. We removed records with unrealistic locations, and geolocated wells to the best of our ability for each county.

For information about data availability and contact information for county-level dataset managers see https://www.sfwmd.gov/doing-business-with-us/permits/well-construction and email: wells@sfwmd.gov

For Broward County, well completion records did not report latitude and longitude information; instead, the township, range and section (PLSS) information was available. We used centroids of these PLSS quadrants as an estimate of the location of the well (accessed February 28, 2018 from ftp://ftp1.fgdl.org/pub/state/). We did not exclude any records on the basis of location.

For Collier, Glades, Hendry, Highlands, Martin, Miami-Dade, Orange, Palm Beach and St. Lucie Counties, we used PLSS (township-range-section) data to geolocate wells instead (see Broward County discussion). We used quarter data, where available; some well completion records did not have quarter data, and were geolocated to the nearest township-range-section centroid.

For Lee County, we geolocated Lee County wells using geospatial data recommended to us by our contact. Geospatial data used to locate wells was downloaded March 1, 2018 from https://leegisopendata2-leegis.opendata.arcgis.com/datasets/b510cf67edfa4891aef56763d7fc440f_0. We did not exclude records on the basis of location.

For Martin County, we excluded 1,501 records (35.6% of dataset) on the basis of township-range-section entries that could not be geolocated.

For Okeechobee County, we excluded 18 records on the basis of unrealistic locations (e.g., offshore or out of state).

For Orange County, we excluded 64 records where we could not geolocate the recorded township-range-section data.

## Hawaii

Hawaii well water level data were sent to us via personal communication (Hawaii's Commission on Water Resource Management; https://dlnr.hawaii.gov/blog/category/commission-on-water-resource-

management). We acknowledge the Commission on Water Resource Management for their helpful responses to our inquiries and for sending data for the state of Hawaii. Five records were removed because their locations appeared implausible (i.e., coordinates over the open ocean).

Data available via personal communication; database manager is Roy Hardy (see: https://www.higp.hawaii.edu/hggrc/projects/hawaii-state-waterwells/)

## Illinois

Illinois groundwater well data were downloaded through personal communications with the Illinois State Geological Survey (http://www.isgs.illinois.edu/ilwater). We acknowledge and thank the Illinois State Geological Survey for their help as we reviewed these well construction records.

Primary contact for well completion and well water level data: Brent Lemke (see https://www.isgs.illinois.edu/about-isgs/staff-directory)

## Indiana

The Indiana groundwater well dataset was provided by the Indiana Department of Natural Resources (https://www.in.gov/dnr/). The following records were excluded on the basis of implausible location data: (i) n=10,242 records have values of "0" listed under UTM, (ii) 4 records have latitudes and longitudes listed under UTM-X and UTM-Y (some cases with negative longitudes, other cases with positive longitudes), (iii) all records with implausibly high or low UTM values outside of state boundaries, and (iv) 237,490 records without any UTM coordinates (blank values). In total, 247,761 records were excluded on the basis of blank or implausible coordinates, summing to ~two-thirds of all records in the dataset. Where UTM coordinates were available, we used these data to geolocate recorded wells in the dataset. Where UTM coordinates were unavailable and where township, range and section data were recorded, we used township-range-section centroids to approximate well locations (township, range and section geospatial data from: http://maps.indiana.edu/previewMaps/Reference/PLSS_Sections.html).

Unfortunately, the Indiana well construction dataset did not provide meridian data for the well records. There are two meridians in the PLSS system for Indiana. The great majority of wells are located in areas spanning the second meridian; however, several counties are covered by the first meridian (Randolph, Jay, Wayne, Union, Franklin, Dearborn, Ohio, Switzerland). For well records stating that the well exists in one of these counties where more than one meridian is possible we did not estimate latitudes and longitudes using township-range-section data. For all other wells located in a county where only one meridian is possible, we estimated locations using township-range-section-quarter information. Where both recorded UTM data and township-range-section-quarter information were available, we found that our estimated locations agreed with the UTM recorded locations within 1 mile for nearly all (98%) of cases (the remaining ~2% of records, where UTM and PLSS coordinates produced locations differing by more than 1 mile, are interpreted as containing errors in either the PLSS data or the UTM coordinates). In total, we excluded 8,685 records where UTM data were unavailable and where township-range-section data were unavailable.

Data available via direct download from https://secure.in.gov/apps/dnr/dowos/AdvancedSearch.aspx

## Iowa

The Iowa groundwater well database was downloaded from GIS Section of the Iowa Geological and Water Survey with the Iowa Department of Natural Resources (https://programs.iowadnr.gov/nrgislibx). The Iowa Geological Survey hosts a separate database entitled "GEOSAM" that is "Iowa's geologic site and sample tracking program" (quote from https://www.iihr.uiowa.edu/igs/geosam/home). Our contact at the Department of Natural Resources sent us a lookup table that allowed us to connect well construction

records to counterpart data stored in GEOSAM. This step is important because the raw well construction database did not contain key information (e.g., static water level and well yield measurements) of interest to our analysis. We acknowledge and thank the Geological and Water Survey with the Iowa Department of Natural Resources for compiling the well construction records and helping us interpret the available data.

Data available via direct download from https://geodata.iowa.gov/dataset/geologic-sampling-points-iowa

## Kentucky

Kentucky data was downloaded from the Kentucky Geological Survey's Water Well Records Search web page (accessed February 16, 2018): http://kgs.uky.edu/kgsweb/datasearching/water/waterwellsearch.asp.

Data available via direct download from http://www.uky.edu/KGS/water/research/gwreposit.htm

## Maine

Maine groundwater well construction information was downloaded from the Department of Agriculture, Conservation and Forestry (http://www.maine.gov/dacf/mgs/pubs/digital/well.htm, accessed November 28, 2017). Two datasets were downloaded: (i) 'mgs_wells.xls' (which contains 62,805 records of 'located wells' that 'could be geographically located' – quotes from the aforementioned web page), and (ii) 'mgs_wells_unlocated.xls' (which contains 63,687 records of 'unlocated wells'). We acknowledge the support of the Maine Geological Survey and thank them for answering questions that helped us interpret the available data.

Data available via direct download from http://www.maine.gov/dacf/mgs/pubs/digital/well.htm

## Maryland

Maryland groundwater well data were provided through personal communication with Maryland's Department of Environment. The data were received via mailed CD and first opened January 2, 2018. The dataset contains 556,213 records. We excluded 114,926 records (21% of all records in the dataset) on the basis of either missing or implausible coordinate data. 441,287 records have recorded locations that are within the boundaries of the state of Maryland; these records were retained for analysis and all others, having either blank coordinates or coordinates corresponding to locations outside the state of Maryland, were excluded.

Data accessed via personal communication with Maryland's Department of Environment (see contact information http://www.doit.state.md.us/phonebook/level2offices.asp?AID=MDE)

## Massachusetts

We downloaded state-level well completion data November 28, 2017 from the Massachusetts Department of Environmental Protection (https://eeaonline.eea.state.ma.us/dep/searchwell). The database contains 191,162 records. We excluded a total of 153,376 records (80.2% of all records) because of either a blank value listed under the location heading or because the locations were outside of state boundaries.

Data available via direct download: https://www.mass.gov/service-details/well-database

## Michigan

Michigan well construction records were downloaded from the Michigan GIS Open Data Portal, provided by the Michigan Department of Technology, Management & Budget. The Michigan dataset provided by the Michigan Department of Technology, Management and Budget contains 325,636 records. At the time of download (February 23, 2016), the data were available at the URL:

www.mcgi.state.mi.us/mgdl/ground_water/Wellogic_Complete_Statewide_Wells/Wells_Complete.zip. A representative from the Michigan state government conveyed the following information about the dataset: *"Wellogic contains primarily water well records from 2000 and newer. So, not all wells installed prior to 2000 are in Wellogic. Many are, but not all. Wellogic contains only "water" wells (household, public, heat pump, industrial, irrigation, test, augmentation, etc.) meaning those wells where groundwater is removed. It does not contain monitoring or injection wells."* We acknowledge and thank the Michigan Department of Environmental Quality for their help amassing these well construction records, and for devoting time to helping us interpret the dataset. We excluded 2,738 records on the basis of implausible well depths.

Data available via direct download: http://www.mcgi.state.mi.us/mgdl/ground_water/Wellogic_Complete_Statewide_Wells/Wells_Complete.zip

## Minnesota

Minnesota groundwater well data were downloaded from the Minnesota Geological Survey. The database contains 464,035 records stored in a series of files that can be linked together by a unique relational identifying number. Our personal contact—a representative from the Minnesota Geological Survey—informed us that "(t)he state of Minnesota enacted legislation in 1974 requiring the submission of well records by well drillers," and that "records for wells drilled before 1974 are spotty at best," and that "We estimate (roughly) that there are perhaps 2.25 million wells in the state" of which only ~20% are recorded in the County Well Index database (herein the Minnesota well database). We acknowledge the Minnesota Geological Survey and thank them for their helping us to interpret and incorporate their well completion database for our analysis.

Data available via direct download from: www.mngeo.state.mn.us/chouse/ground_water/gis_data.html

## Mississippi

Mississippi groundwater well data were provided through personal communication with the Water Resources Management Division of the Office of Land and Water Resources in the Mississippi Department of Environmental Quality. The database contains 158,844 records. We acknowledge and thank the Water Resources Management Division of the Office of Land and Water Resources in Mississippi's Department of Environmental Quality for providing access to groundwater well construction data, and for working with us through multiple discussions to help us correctly interpret the available data.

Data accessed via personal communication; see https://www.mdeq.ms.gov/about-mdeq/contact-mdeq/staff-directory/ and contact Sandra Dowty for well completion data

## Missouri

The Missouri groundwater well data were downloaded from the Missouri Spatial Data Service (http://msdis.missouri.edu) in March of 2016. We surveyed and discussed the data with a representative from the Missouri Geological Survey in September of 2017. The database contains 227,717 records. We acknowledge the Missouri Department of Natural Resources' Missouri Geological Survey for their help with our research.

Data available via direct download from http://www.msdis.missouri.edu/index.html

## New Hampshire

We were granted access to New Hampshire groundwater well construction data through personal communication with the New Hampshire Geological Survey (New Hampshire Department of Environmental Services). We first downloaded the data for the state on December 15, 2017. The dataset contains 130,843 records. Location information stored in the columns entitled 'LATITUDE' and 'LONGITUDE' was recorded in a number of formats. For example, some of the records were likely stored in degrees-minutes-seconds formats, whereas others were stored as degrees-minutes-decimal minutes ("." values were used for all delimitations). We interpreted these location data to the best of our ability, but acknowledge the possibility that a (likely small) fraction of these records contain transcription errors (i.e., misinterpretation of the originally-recorded latitude and longitude data). Overall, we were able to interpret latitudes and longitudes for 63,274 records (48.4% of all records); i.e., we excluded 67,569 records because of blank or implausible well location values. We excluded a further 5,591 records because their locations lie outside the state of New Hampshire. In total, we excluded 73,160 records on the basis of their recorded location data (55.9% of all records in the dataset).

Data available via personal communication; see www.des.nh.gov/organization/commissioner/gsu/wwip/index.htm and contact Gregory Baker

## New Jersey

New Jersey well data were sent to us via personal communications with the Bureau of Water Allocation and Well Permitting. We obtained two datasets: one consisting of 20,543 records on January 26, 2018, the second consisting of 187,893 records on March 22, 2018. In total, we analyzed 208,436 records. We excluded a total of 1,043 records (0.5% of all records) because of either a blank value listed under the location heading or because the locations were outside of state boundaries.

Primary contact information for database manager: email: wellpermitting@dep.nj.gov

## New York (1/2): Outside of Long Island

We downloaded state-level well completion data via personal communications and a Freedom of Information Law request through the New York Department of Environmental Conservation. The database contains 114,220 records; the Freedom of Information Law Request was granted (i.e., the data was made available to us) on December 6, 2017. We excluded a total of 19,579 records (17.1% of all records) because of either a blank value listed under the location heading or because the locations were outside of state boundaries.

Primary contact for well completion data is Beth Guidetti (see https://www.dec.ny.gov/lands/33317.html)

## New York (2/2): Long Island

We downloaded New York state-level well completion data for the Long Island region through communications with the Department of Conservation. Data was provided through a Freedom of Information Law Request on January 31, 2018. The database contains 50,464 records. We excluded a total of 46,208 records (91.6% of all records) because the records did not contain latitude and longitude data. We excluded a further 172 records with locations that fell outside of the state of New York's outermost terrestrial boundary. In sum, we excluded 46,380 records on the basis of their locations, summing to 91.6% of all records in the New York – Long Island dataset.

Primary contact for well completion data is Beth Guidetti (see https://www.dec.ny.gov/lands/33317.html)

## North Carolina

North Carolina groundwater well data were provided through personal communication with North Carolina's Department of Environmental Quality - Division of Water Resources on February 13, 2018. The dataset contains 253,310 records. Of the 253,310 records, 215,409 records (85.1% of all records) do not have a latitude or longitude value recorded in the North Carolina groundwater well construction dataset. This large proportion (85.1%) of records poses a complication, as we did not wish to exclude such a large percentage of records. For other states' groundwater well construction datasets we have used alternative geolocation approaches to estimate well latitude and longitude values (e.g., Indiana and Alaska, where township-range-section data were used to locate wells). The North Carolina well database does not contain PLSS information; it does contain addresses for some ~45,000 records (i.e., fields entitled 'FACILITY_ADDRESS' and 'FACILITY_LOCATION'), ~one-quarter of which follow the format [Street Number (e.g., "4309")] [Street Name] (e.g., "Spring Lawn") [Street Type (e.g., "Rd")]. Nevertheless, many of these addresses could not be matched easily with geocoded addresses for the state of North Carolina. We therefore recognize that more wells could possibly be geolocated for this state dataset, but we did not do so for this analysis, instead moving forward to analyze only wells with latitude-longitude coordinate data. In addition to the large share of well records excluded on the basis of missing well location data (85.1% of all records), we excluded an additional 3,328 records (1.3% of all records) with latitude and longitude pairings that fall outside of North Carolina's boundaries.

For primary contact information for database manager see communication; https://deq.nc.gov/about/divisions/water-resources/water-resources-permits/wastewater-branch/ground-water-protection/well-program

## Rhode Island

Rhode Island groundwater well completion data were provided through personal communication with Rhode Island's Department of Health on December 1, 2017; the dataset itself is from a local well completion company that has operated throughout the state for more than five decades (company is entitled 'JASWELLS'). The JASWELLS database contains 1,928 records; unfortunately, no water level data were available in the dataset.

Primary contact for well completion data inquiries is Shannon Harrower-Nakama (Center for Drinking Water Quality, Rhode Island Department of Health, 3 Capitol Hill, Providence, Rhode Island).

## South Carolina (1/2): Coastal Plain

We downloaded well completion data via personal communications with the South Carolina Department of Natural Resources on November 28, 2017. This dataset represents one part of two databases sent to us (see subsequent section for the second dataset). This dataset focuses on counties in the South Carolina Coastal Plain. The database contains 13,781 records. We excluded a total of 23 records (0.2% of all records) because the locations were outside of state boundaries (e.g., location is just offshore).

Primary contact for well completion data is Andy Wachob (see www.dnr.sc.gov/water/hydro/staff.html)

## South Carolina (2/2): Piedmont

We downloaded state-level well completion data November 28, 2017 via personal communications with the South Carolina Department of Natural Resources. This dataset is the second of two parts for South Carolina, covering counties located in the northwestern portion of the state. The database contains a total of 26,936 records. We excluded 2,822 records on the basis of blank or implausible well location information (e.g., location outside of state boundaries). In all, these 2,822 excluded records sum to 11.3% of all records in the database.

Primary contact for well completion data is Andy Wachob (see www.dnr.sc.gov/water/hydro/staff.html)

## Vermont

Vermont groundwater well construction information was downloaded from the Agency of Natural Resources Open Data Portal (http://anrgeodata.vermont.gov/datasets?q=wells). We downloaded multiple files from the webpage entitled (i) ‘Private wells’, and (ii) ‘Public Water Sources’ and merged these individual datasets. The data were first accessed November 28, 2017, and we worked with a contact at the Department of Environmental Conservation to best interpret the available data. The database contains 107,648 records. We acknowledge the Vermont Agency of Natural Resources’ Department of Environmental Conservation for their help amassing the data and for helping us to best interpret the data. We excluded 111 records with recorded locations falling outside Vermont’s state boundaries (corresponding to 0.1% of all records for Vermont).

Data available for direct download: http://anrgeodata.vermont.gov/datasets?q=wells

## Virginia

We accessed state-level well completion data via personal communication with the Virginia Department of Environmental Quality. The Virginia well completion dataset contains 66,572 records and was first accessed by us on December 21, 2017. A large fraction (93.8%) of records do not contain construction dates and were excluded from our analysis. We also excluded 4,381 records with blank or unreasonable well locations (6.6% of all 66,572 well construction records for the state of Virginia’s well completion database).

For contact information for dataset manager see: www.deq.virginia.gov/Programs/Water/WaterSupplyWaterQuantity/GroundwaterProtectionSteeringCommittee/WellheadProtection.aspx

## Wisconsin

Wisconsin groundwater well data representing records constructed after the year 1988 were exported from a CD sent to us by the Wisconsin Department of Natural Resources in spring 2016. Data do exist for wells constructed prior to 1988 in a separate database that contains 354,014 records; unfortunately, the only data available in digitized and tabulated format of interest are the well latitudes and longitudes themselves, most of which have not been field-verified (pers. comm. with representative from the Wisconsin Geological Survey). We did not include the dataset focusing on wells constructed prior to 1988 for this analysis. We geocoded wells based on their township-range-section-quarter (PLSS) data. Locations are therefore approximations to ~1 mile or better. We only geocoded records with at least township-range-and-section data; where quarter, quarter-quarter data were available, we used these to refine our estimates of longitude and latitude based on centroids of township-range-section grids.

Data available for direct download via prodoasext.dnr.wi.gov/inter1/spinvent$.startup

**Note 2: Accuracy of elevations extracted by geospatial analyses –** Comparison of recorded elevations of monitoring wells versus digital elevation geospatial data

## Note 2. Accuracy of elevations extracted by geospatial analyses

We compared elevations of land surfaces at wellheads derived from (i) digital elevation data extracted at each recorded latitude/longitude pairing for each monitoring well, and from (ii) land surface elevations for the same wells, as recorded in the United States Geological Survey's (USGS') monitoring well database. The elevations extracted from the 10 m by 10 m digital elevation model (NED 1/3 arc second data from: http://ned.usgs.gov) compare closely to those recorded in the United States Geological Survey's database (Supplementary Figure 2), strengthening our confidence in the precision of USGS monitoring well locations.

An unknown but non-zero fraction of the USGS' reported well locations or land surface elevations are incorrect. These imperfections in the USGS database contribute to the observed mismatches in land surface elevations shown in Supplementary Figure 2.

For example, USGS site "USGS 471639122132001 21N/05E-30L05" presents a land surface elevation of "9,999 feet above NAVD88" (as do other USGS sites, including: "USGS 441500106415001 49-082-02bb 01"). While this is almost certainly an indicator for a no value entry, its inclusion in the USGS' database risks misinterpretation, especially because land surface elevations that are actually 9,999 feet above sea level exist in the United States, meaning some of these values could be real while others may not be.

As another example, USGS site "USGS 481845118020001 30N/37E-06Q02" states the following "Land surface altitude: 1,800 feet above NGVD29." (https://waterdata.usgs.gov/nwis/inventory/?site_no=481845118020001&agency_cd=USGS& accessed November 27, 2019). But, entering these coordinates into The National Map Viewer yields the following: "Elevation: 3320.97 Feet; Source: 3DEP 1/3 arc-second" (https://viewer.nationalmap.gov/theme/elevation/##bottom accessed November 27, 2019).

Were the USGS well location and land surface elevation data more reliable, it is likely that the match between the digital elevation model value at each well location and the recorded USGS well land surface elevation (i.e., Supplementary Figure 2) would agree more closely.

**Note 3: Quality assurance of constructed well water level data –** Comparison of water levels in monitoring wells versus constructed wells

## Note 3. Quality assurance of constructed well water level data

Most of the well water level data we present originate from 'static water levels' recorded in well completion reports. We note that that some of these measurements may not reflect actual conditions in the aquifer system, potentially because the drilling process impacted local hydraulic heads, or because not all measurements were made by highly trained technicians (e.g., as assumed to be the case for measurements reported by the United States Geological Survey (USGS) and California's Groundwater Ambient Monitoring and Assessment Program (GAMA)).

Therefore, before proceeding with our analysis, we explored the quality of the constructed well water levels. Our test assumes that USGS and GAMA measurements (i.e., monitoring well water level measurements) accurately reflect hydraulic heads at the monitoring well location, and in the immediately adjacent aquifer. Our evaluation required a series of geospatial analyses, each completed in order to improve the likelihood that the constructed-versus-monitoring well water levels are measuring similar hydrogeologic conditions (so that they may be compared for consistency). These steps – detailed below (Supplementary Figure 3) – include:

- (*i – similar locations*) comparing water level measurements made in monitoring wells and constructed wells with similar latitudes and longitudes, to exclude comparisons of wells located far from one another;
- (*ii – similar elevations*) comparing water level measurements made in monitoring wells and constructed wells with similar elevations, to exclude comparisons where topographic gradients are high;
- (*iii – similar depths)* comparing water level measurements made in monitoring wells and constructed wells with similar depths, to exclude comparisons of wells screened at different depths that may capture different hydraulic heads;
- (*iv – similar measurement date*) comparing water level measurements made in monitoring wells and constructed wells at similar times, to exclude comparisons that are susceptible to changes in groundwater levels over time

*(i – similar locations)* We first identified monitoring wells and constructed wells that are located within 1.61 km (1 mile) of one another. We found 578,058 monitoring wells that are located within 1.61 km of at least one constructed well, and 27,763,281 constructed wells that are located within 1.61 km (~1 mile) of at least one monitoring well.

(*ii – similar elevations*) Next, we filtered these pairs of monitoring and constructed wells to include only those with land surface elevations within ±1 m of one another. Any compared constructed and monitoring wells with zero values for their estimated elevation (e.g., located slightly offshore) were excluded.

(*iii – similar depths)* Next, we further filtered the pairs of monitoring and constructed wells to include only those with well depths that are within ±1 m of one another. Any compared constructed and monitoring wells where one of the two wells does not have a recorded well depth value were excluded (e.g., we excluded cases where the monitoring well depth was unknown).

Some state- and regional-scale well construction databases also record the construction of monitoring wells (e.g., see well completion purposes for 17 western states reported in Perrone, D. & Jasechko, S. Dry groundwater wells in the western United States. *Environmental Research Letters* **12,** 104002 (2017)). To improve the likelihood that our comparison of constructed-versus-monitoring well water levels represents a comparison of water levels in two different wells, we excluded all cases where the recorded well depth for the constructed and monitoring wells were identical (recorded well depths must differ by >10 cm).

(*iv – similar measurement date*) Lastly, we only used well water level measurements reported in well completion reports with well construction dates that are within ±1 week (Supplementary Figure 5) and ±1 month (Supplementary Figure 4) of a well water level measurement in the corresponding monitoring well (i.e., the well construction date and the monitoring well water level measurement data are separated by no more than ~1 month).

To further reduce the possibility that our comparison represents well water level measurements made in the same well, we removed any cases where the recorded constructed well date and monitoring well water level measurement date were within one day of one another. These steps (i - iv) are depicted in Supplementary Figure 3.

Our comparison demonstrates the robustness of constructed well water level measurements. Under our most strict set of criteria for comparing constructed-versus-monitoring well water level measurements (constructed and monitoring wells are: (i) within one mile, (ii) have land surface elevations within 1 m, (iii) have depths within 1 m, and (iv) have measurements made within one week of one another), we show that nearly all (>95%) water level measurements agree within ±3 m (Supplementary Figure 5).

Relaxing our time criteria and comparing well water level measurements made within one month (30 days) of one another, we find that the correspondence between constructed and monitoring well water levels is still strong, but slightly weaker than our comparison of measurements made within one week of one another (Supplementary Figure 4). Some of this additional scatter may arise because of actual temporal variations in groundwater levels during the time interval between the monitoring well water level measurement and the constructed well water level measurement.

**Note 4: Sensitivity of our results to the selected distance to the coast and well depth –** How the maximum distance from the coast and well depth impacts our results

## Note 4. Sensitivity of our results to the distance to the coast and well depth

**Sensitivity of our results to the selected distance to the coast**

We show that the fraction of well levels lying below sea level varies with distance to the coast (Figure 3 main text). It is therefore plausible that our results are sensitive to the selected distance from wells to the coast used to determine segments of coastline where at least half of all well water levels lie below sea level. The discussion in the main text (and Figure 4 main text) uses a distance threshold of 10 km (i.e., well water level measurements made within 10 km of the coast are used to determine which 20 km segments of coastline are likely to have landward hydraulic gradients).

Here we assess the sensitivity of our results to the selected distance to the coast. We repeat our analysis for two more distance thresholds: (i) using only well water level measurements made within 5 km of the coast, and (ii) using only well water level measurements made within 2 km of the coast. The results of these sensitivity analyses are tabulated in Supplementary Table 1.

Overall, our main findings, reported for a distance threshold of 10 km (i.e., those reported in the main text and Figure 4 main text), remain largely unchanged when we consider alternative thresholds: 5 km and 2 km (Supplementary Table 1); if anything, our results presented in the main text understate the prevalence of landward hydraulic gradients as evidenced by the tabulated results for 5 km and 2 km well-to-coast distance thresholds, which suggest these landward hydraulic gradients may be *more* abundant than we report in the main text (Supplementary Table 1; Supplementary Figures 6-8).

**Sensitivity of our results to the selected threshold maximum well depth**

Non-zero vertical hydraulic gradients are expectedly widespread around the globe. One implication of non-zero vertical hydraulic gradients is that deeper and shallower wells in a similar x,y location can have different well water level elevations. Further, deeper wells tend to be more likely to capture confined aquifer conditions, implying the inclusion of shallower versus deeper wells in our analysis may impact the aquifer conditions we assess.

Here we tested the sensitivity of our results to well depth. We do so by re-running our analyses using a suite of threshold depths—namely, a maximum well depth value that a study well must be shallower than in order to be included in our analyses. We varied these well depth thresholds as follows:

(i) results using only water level measurements made in wells shallower than 25m (compared against results derived solely from wells deeper than 25m) – Supplementary Figure 9.

(ii) results using only water level measurements made in wells shallower than 30m (compared against results derived solely from wells deeper than 30m) – Supplementary Figure 10; this 30 m threshold is shown for consistency with the vertical scale studied by Sawyer, A. H., David, C. H., & Famiglietti, J. S. Continental patterns of submarine groundwater discharge reveal coastal vulnerabilities. Science, 353, 705-707 (2016).

(iii) results using only water level measurements made in wells shallower than 50m (compared against results derived solely from wells deeper than 50m) – Supplementary Figure 11

(iv) results using only water level measurements made in wells shallower than 75m (compared against results derived solely from wells deeper than 75m) – Supplementary Figure 12

(v) results using only water level measurements made in wells shallower than 100m (compared against results derived solely from wells deeper than 100m) – Supplementary Figure 13

(vi) results using only water level measurements made in wells shallower than 200m (compared against results derived solely from wells deeper than 200m) – Supplementary Figure 14

Water levels that lie below sea level tend to be more common for wells deeper than a threshold well depth, compared to their prevalence in wells shallower than a threshold well depth (i.e., more orange colored bars in the lower parts of Supplementary Figures 9-14 relative to the upper plots in Supplementary Figures 9-14). Our finding is consistent with the existence of downward-oriented vertical hydraulic gradients.

We find that well water levels that lie below sea level are more common in deeper wells (Supplementary Table 2). For example, see the final row of Supplementary Table 2, where only wells deeper than 200 m are analyzed (and a majority of well water levels lie below sea level in 44.4% of analyzed segments, compared to just 2.6% of wells shallower than 200 m). Part of this discrepancy arises because of the small share of coastal segments that have a sufficient number of wells deeper than 200 m for us to analyze (see Supplementary Figure 14 and compare top versus bottom panels). Yet we also find that the general spatial patterns of segments of coastline where most well water levels lie below sea level holds across a wide range of threshold well depths (Supplementary Figures 9-14).

## Note 5: Density of monitoring well network versus driller report water level dataset

## Note 5. Density of monitoring well network versus driller report dataset

Our work is based on two data sources: (i) well water level data reported in USGS and GAMA monitoring wells, and (ii) water level data derived from well completion reports. Here we compare the density and results obtained from each dataset. First, we tabulate the number of unique wells reporting at least one water level measurement after the year 2000 among the different datasets. We show that the USGS (and GAMA) dataset—while providing important and locally relevant insights where these wells do exist—is far too sparse to provide useful insights along the vast majority of the U.S. coastline. Specifically, for example, the analyzed USGS data for the entire Gulf Coast has only 706 monitoring wells reporting at least one water level within 10 km of the coast; by contrast, the compiled well completion report water level dataset contains 141,426 post-2000 measurements each in unique wells along the Gulf Coast—200 times the density of the USGS' network. The driller report water level dataset is 20 times denser than the USGS' network along the East Coast, and 6 times denser than the combine USGS/GAMA network along the West Coast (calculated as the number of unique wells with at least one post-2000 water level measurement that exist within 10 km of the coast; see the first row reporting values in Supplementary Table 3). Thus, including the drillers report dataset greatly increased the data density and the statistical significance of our analyses.

Second, we re-ran our analyses (i.e., as in Figure 4 in the main text) using (A) only water levels from drilling reports, and (separately) (B) only water levels from USGS/GAMA monitoring wells. We apply the same requirements for analyses that we do elsewhere in our study (i.e., at least ten water level measurements made in unique wells binned to the nearest 20 km-long coastline segment). The results are presented in Supplementary Figure 15, and highlight the much higher number of 20 km-long segments meeting our criteria for analysis when we use only the driller report data (compared to the sparse set of segments with sufficient USGS/GAMA monitoring water level data for us to analyze). Our results focus solely on water levels measured within 10 km of the coast (Supplementary Figure 15).

**Note 6: Impact on results of imprecise latitude-longitude data for California –** How imprecisions in California constructed well latitude-longitude data impacts results

## Note 6. Impact on results of imprecise latitude-longitude data for California

Well locations (i.e., latitude-longitude pairings) are generally geocoded to the centroid of the nearest township-range-section in California's groundwater well completion database (Supplementary Figure 16). This imprecision leads to uncertainty in well water level elevations, because we extract land surface elevations from a digital elevation model, and because elevations can vary considerably within ±1 mile (1.6 km), the approximate length and width of a township-range-section quadrant. We assess how uncertainty in wellhead locations may affect our conclusions about the abundance of well water levels that lie below sea level.

To evaluate the sensitivity of our results to uncertain wellhead elevations, we calculated the maximum and minimum elevations within each township-range-section for constructed wells located within ±1 meter of the centroid of these quadrants (implicitly assuming that wells geocoded to locations >1m from the centroid of a township-range-section are accurately geocoded, representing the real location of the well rather than the centroid of the township-range-section quadrant that the well lies within). The results of our sensitivity analysis are displayed in Supplementary Figure 17 and Supplementary Table 4. Our broad conclusions—that landward hydraulic gradients are less common along the West Coast relative to the East and Gulf Coasts—remain largely unchanged under either sensitivity scenario (Supplementary Figure 17).

**Note 7: Compilation of previous works reporting coastal hydraulic gradients**

## Note 7. Local-scale studies of seawater intrusion and hydraulic gradients

Here we present a compilation of many studies that report on the following statuses of coastal hydrogeologic conditions: (i) seaward hydraulic gradient (i.e., coastal well water levels above sea level), (ii) landward hydraulic gradient (i.e., coastal well water levels below sea level), and (iii) seawater intrusion (encroachment of seawater inland leading to the salinization of coastal aquifers). These compiled studies are presented in Supplementary Table 5.

## Note 8: Comparison with previous local-scale results

## Note 8. Comparison with previous local-scale studies

Our research demonstrates the existence of landward hydraulic gradients along each contiguous US coastline, building on previous local-scale research. Data compiled and presented in Supplementary Table 5 are displayed in Figure 1 in the main text. We compared our well water level elevation observations against these compiled studies. Our comparison was made by (1) developing a 10 km buffer around each compiled study location from Supplementary Table 5 (i.e., a 10 km buffer around locations where previous research has reported a landward or seaward hydraulic gradient), and (2) presenting the fraction of all well water level observations in our dataset that are within 10 km of a study locations (from Supplementary Table 5) that have a well water level that is below sea level. We analyze all locations where a value of "Yes" is entered in column 7 of Supplementary Table 5 (entitled "Include in Supp. Fig. 19 ?").

We show that the majority of well water levels are above sea level in most of the areas where previous research has suggested a seaward hydraulic gradient exists (i.e., where submarine groundwater discharge has been reported; Supplementary Table 5a), our data show that most well water levels are above sea level within 10 km of these locations). In areas where seawater intrusion or a landward hydraulic gradient has been reported (i.e., see Supplementary Table 5b), we find that many compiled well water levels are below sea level.

One outlier point is the submarine groundwater discharge reported at the Salinas River in California (see rightmost blue square in Supplementary Figure 19). Seawater intrusion is known to take place in this area, as stated by the authors in this work (Lecher, A. L., Fisher, A. T., & Paytan, A. (2016). Submarine groundwater discharge in Northern Monterey Bay, California: Evaluation by mixing and mass balance models. Marine Chemistry, 179, 44-55 – for further evidence for landward gradients see also Figure 5-2 in: https://svbgsa.org/wp-content/uploads/2019/03/Valley-Wide-Integrated-Sustainability-Plan-optimized.pdf). The existence of submarine groundwater discharge proximate to a region where seawater intrusion is known to occur highlights the complexity and spatial variability of coastal hydraulic gradients. We emphasize that seawater intrusion and submarine groundwater discharge are not mutually exclusive at the spatial scale we analyze. Further, the spatial scale at which we complete our analyses (e.g., 10 km inland from the coast and 20 km-long coastline segments) may include places characterized by seaward hydraulic gradients and some other places characterized by landward hydraulic gradients.

Broadly, our comparison of primary well water observations and previous research (i.e., Supplementary Figure 19) demonstrates that the well water level elevation data we present here replicate most previous assessments; that is, locations where hydraulic gradients have been determined to be seaward (blue squares in Supplementary Figure 19) are usually surrounded mostly by well water levels that are above sea level (i.e., blue squares below 50% on y-axis and are far more common on the left side of the plot). Further, the well water level observations capture most of the areas where landward hydraulic gradients exist (i.e., red squares are far more common on the right side of Supplementary Figure 19). We emphasize that the overwhelming majority of locations where seawater intrusion has been identified also have at least some nearby well water levels that are above sea level (i.e., only one location where seawater intrusion has been identified is surrounded entirely by well water levels that are below sea level: "southern Louisiana" on right side of Supplementary Figure 19). This highlights how seawater intrusion can take place even when well water levels are above sea level, plausibly (i) due to the density differences between seawater and freshwater that make coastal aquifers vulnerable to seawater intrusion even when their hydraulic heads are above sea level, or (ii) locally perched rain-fed aquifers below some individual wells.

## Note 9: Well level variations over time observed in coastal monitoring wells

## Note 9. Well level variations over time observed in coastal monitoring wells

Here we explore well water level variations over time in monitoring wells located within 10 km of the contiguous US coast. We analyze monitoring well water level data from the United States Geological Survey and California's 'GAMA' databases. The step-by-step data downloading and quality-control procedures that we adhere to are detailed in full in the supplementary information section S36.2 on pages 125-127 of Perrone, D., & Jasechko, S. Deeper well drilling an unsustainable stopgap to groundwater depletion. *Nature Sustainability* **2,** 773-782 (2019).

First, we identified monitoring wells within 10 km of the contiguous US coast. Next, we mapped and plotted the Spearman rank correlation coefficient for well water level variations from 2000 to 2015. We only present results for monitoring well water level time series that meet all of the following criteria: (1) at least one water-level measurement reported in the first five years of the analyzed time interval (between January 1, 2000 and December 31, 2004), (2) at least one water-level measurement in the final five years of the analyzed time interval (between January 1, 2010 and December 31, 2014), and (3) at least ten water level measurements reported within the time interval 2000-2015.

In total, 1,951 monitoring wells met these criteria: 1,434 monitoring wells within 10 km of the East Coast, 274 within 10 km of the Gulf Coast, 243 within 10 km of the West Coast. We calculated Spearman rank correlation coefficients of well water level versus measurement date. Positive values are consistent with a decline in well water levels over time (i.e., increasing depth to water—deepening of well water levels—over time). Negative values are consistent with increases in well water levels over time (i.e., decreasing depth to water—shallowing of well water levels—over time).

We considered analyzing well water total dissolved solids data, as these data are widely available across the United States (via Qi, S.L., and Harris, A.C., 2017, Geochemical Database for the Brackish Groundwater Assessment of the United States: U.S. Geological Survey data release, https://doi.org/10.5066/F72F7KK1. https://www.sciencebase.gov/catalog/item/583dfd9ee4b088b77f520d07). Analyzing groundwater salinity in coastal areas as a simple function of distance to coast could risk given the misleading impression that the dominant solute source in saline coastal aquifers is seawater; this would be an oversimplification. Work by many authors (e.g., Chowdhury, A. H., Scanlon, B. R., Reedy, R. C., & Young, S. (2018). Fingerprinting groundwater salinity sources in the Gulf Coast Aquifer System, USA. Hydrogeology Journal, 26, 197-213) has highlighted that coastal aquifer salinity can derive from many sources and processes other than seawater intrusion (e.g., evaporite dissolution, sea spray aersols, evapoconcentration). We therefore focus our analysis to well water level observations rather than analyzing groundwater quality data.

**(B) Supplementary Figures**

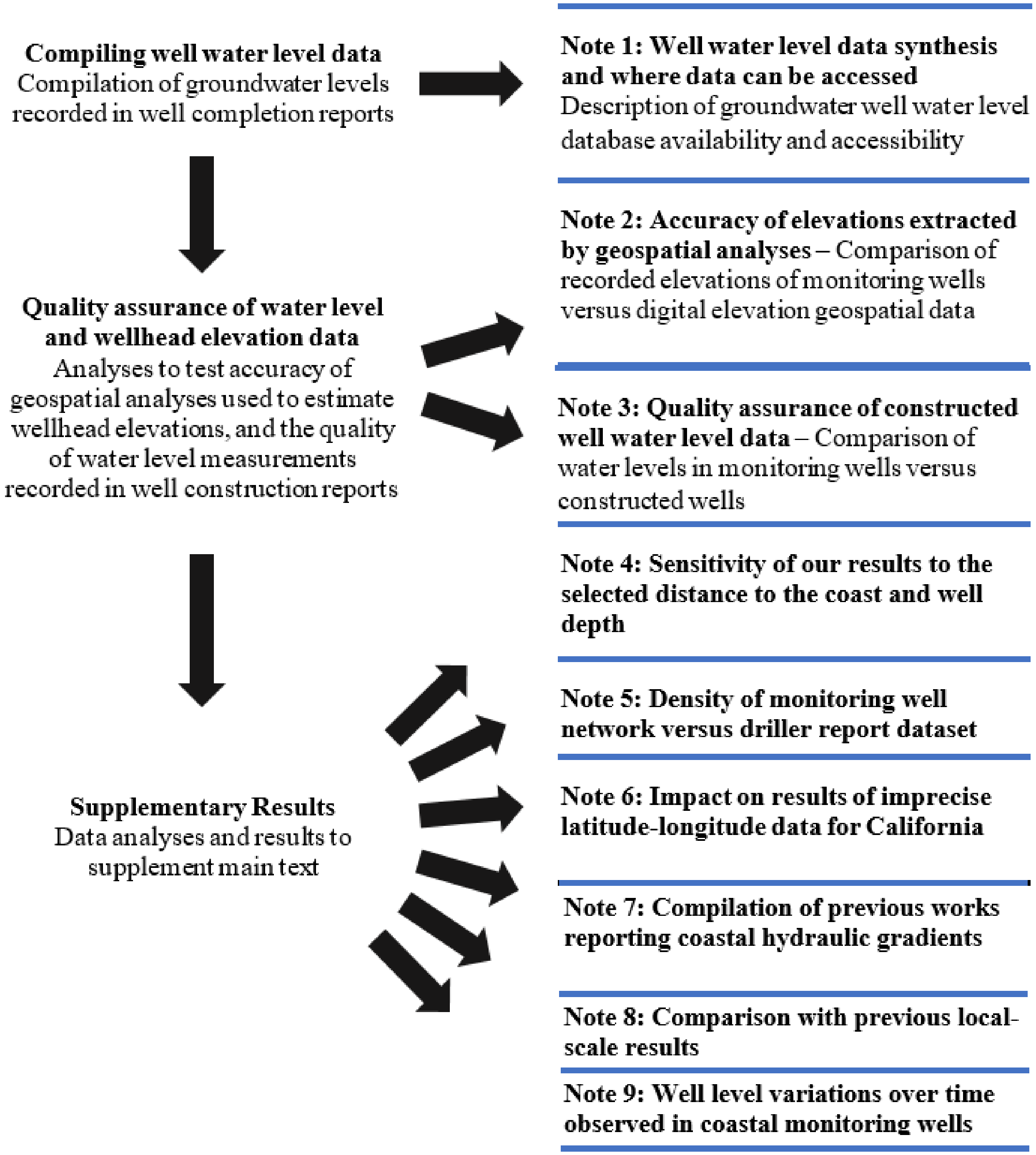


**Supplementary Figure 1.** Layout of Notes in the Supplementary Information.

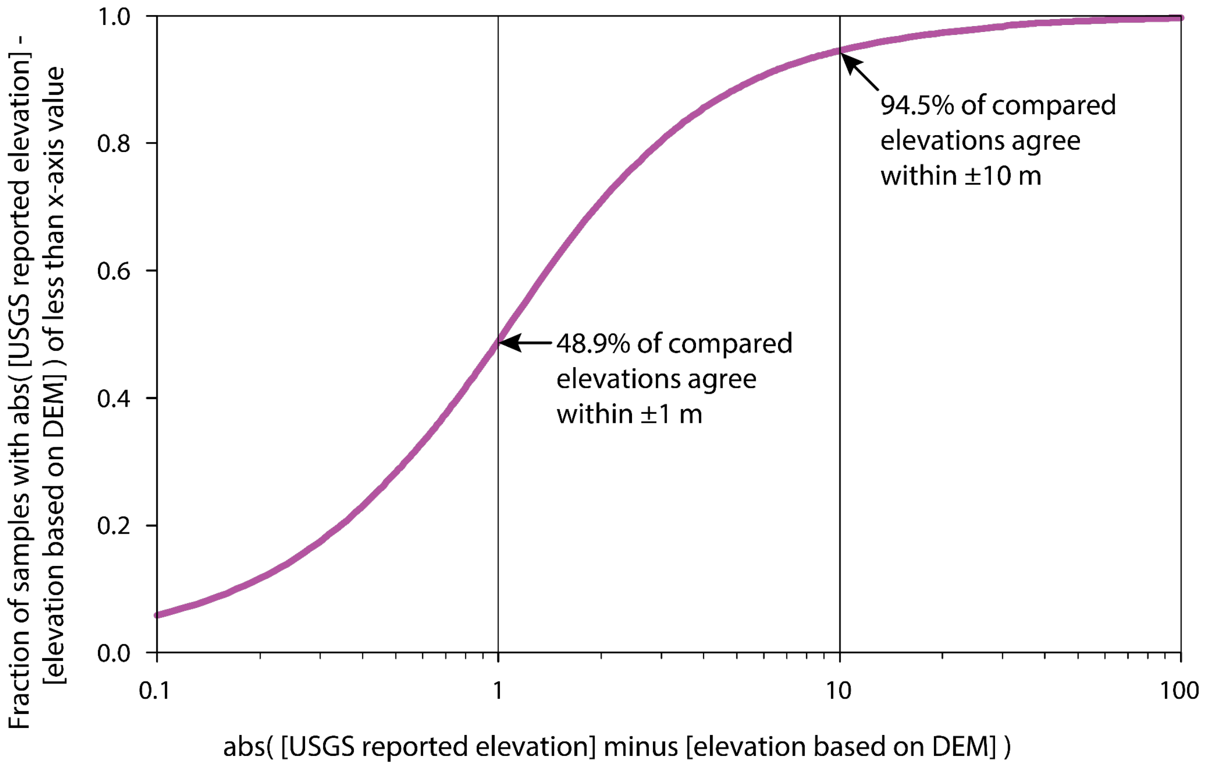


**Supplementary Figure 2.** Comparison of elevations extracted from a digital elevation model (DEM) versus those recorded in the monitoring well databases (i.e., United States Geological Survey's (USGS's) groundwater monitoring well network). The pink line shows the fraction (y-axis) of all compared elevations (i.e., DEM-based versus USGS-based elevations for monitoring wellheads, x-axis) that agree within a certain range (x-axis). For example, the vertical line at an x-axis value of "1" shows that 48.9 % of DEM- and USGS-based elevations agree within 1 m. Further, nearly all compared USGS- versus DEM-elevations agree within 10 m.

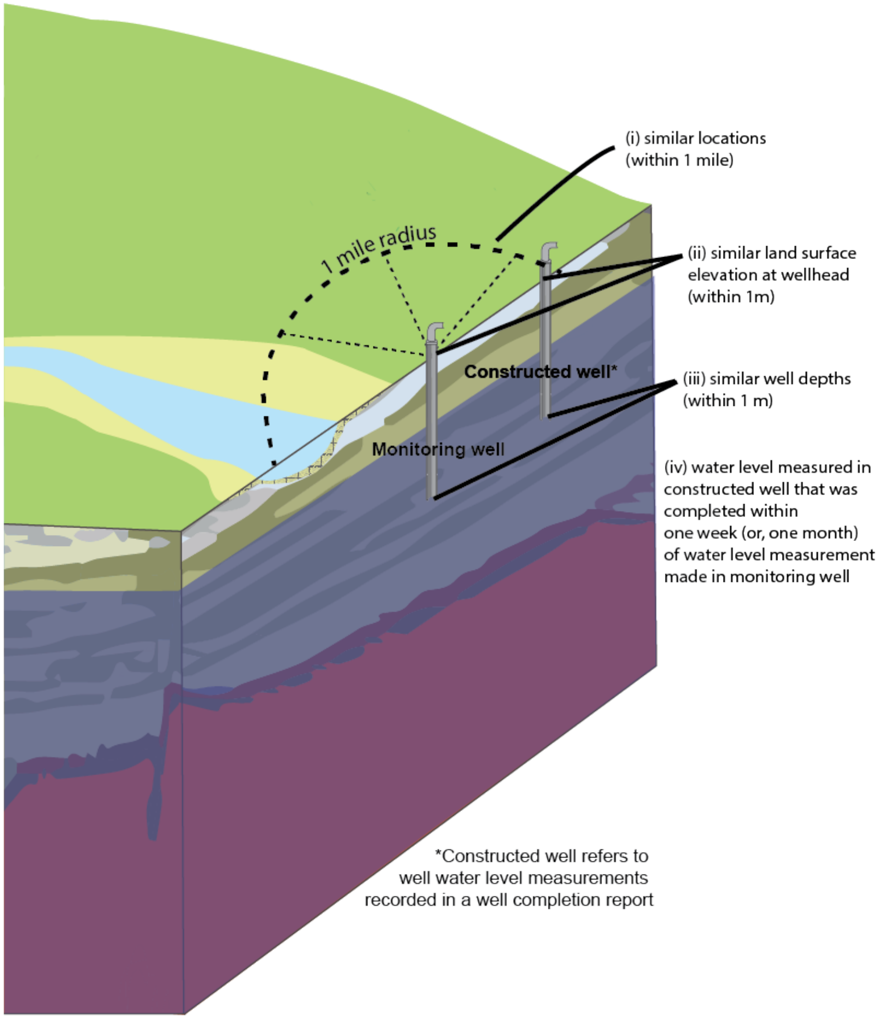


**Supplementary Figure 3.** Conceptual diagram displaying criteria applied in our comparison of constructed well water levels versus monitoring well water levels. The results of our comparisons are shown in Supplementary Figures 4-5.

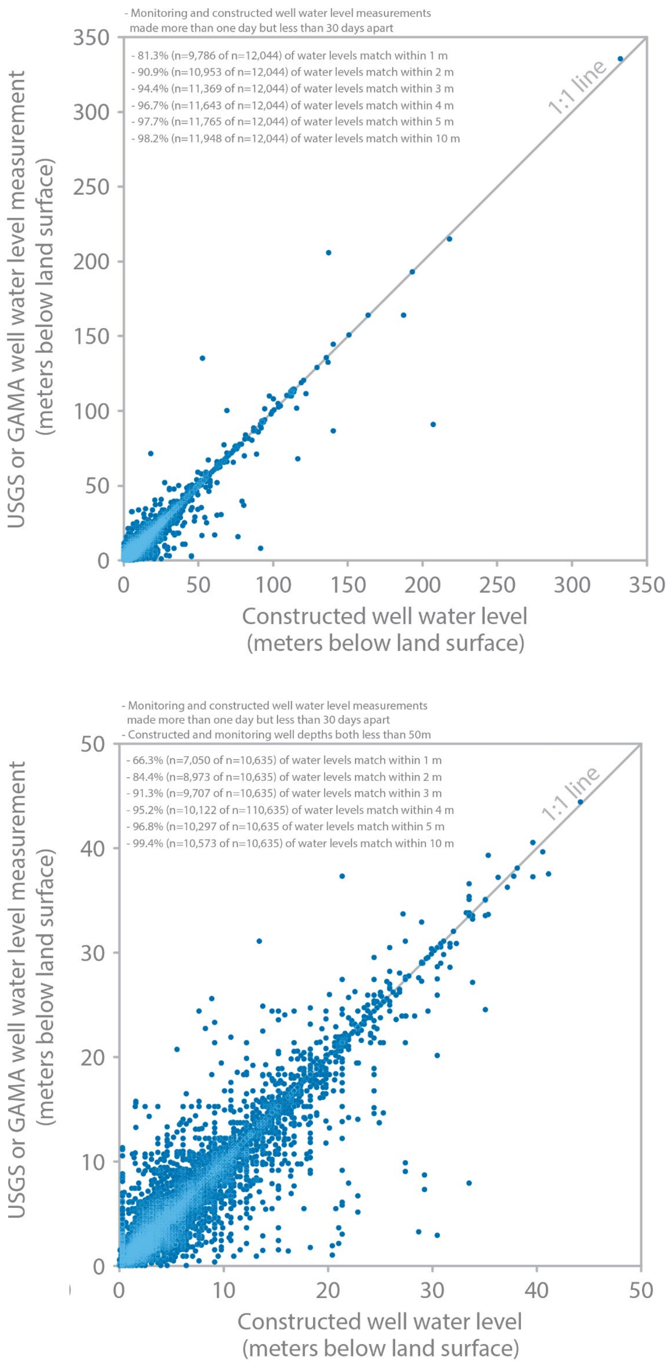


**Supplementary Figure 4.** Comparison of well water levels in constructed and monitoring wells (measurements made within one week of 30 days of one another). Each point represents a monitoring well water level measurement (y-axis) and a driller reported static water level measurement (x-axis) for two wells that are located close together, at similar depths, have similar land surface elevations at their tops, and have measurements made within 30 days of one another (but more than one day apart). We outline each point in light blue so that the density of partially-overlapping points can be interpreted more easily. e top panel presents all points with water levels within 350 m of the land surface (an interval including nearly every point in the dataset); the lower panel presents all points with water levels within 50m of the land surface (i.e., the lower plot represents a "zoom in" of the lower-left corner of the top panel).

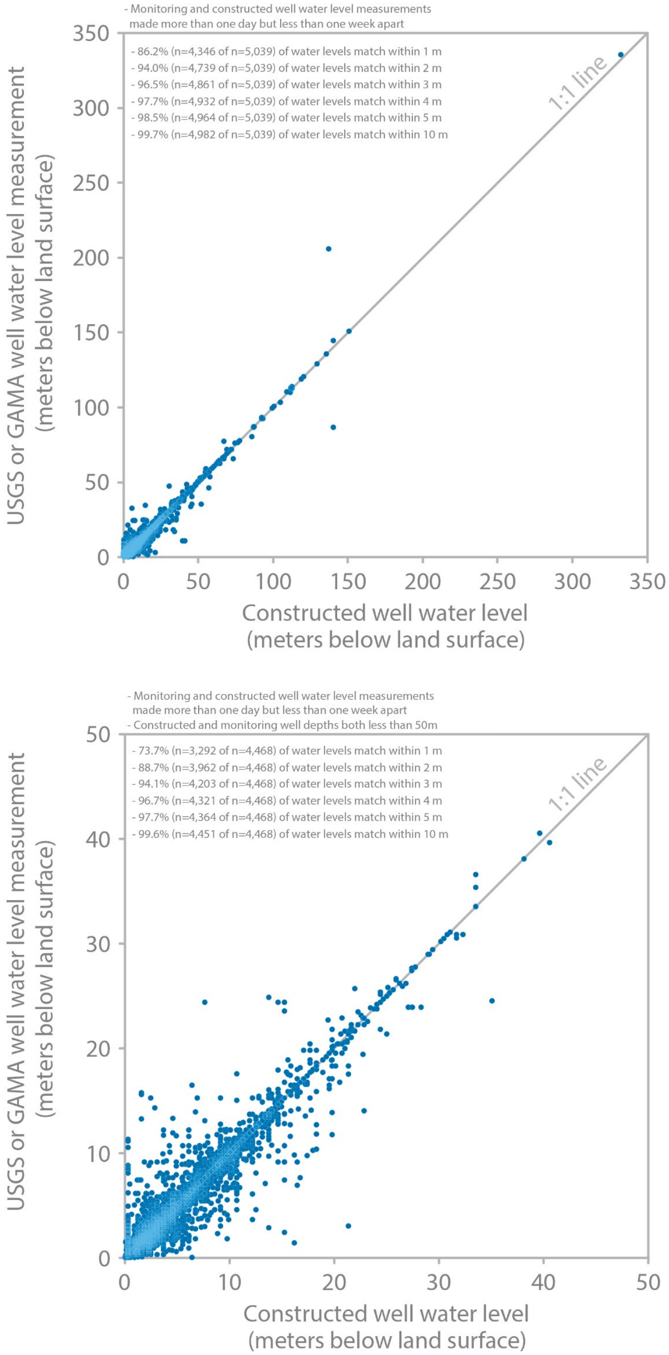


**Supplementary Figure 5.** Comparison of well water levels in constructed and monitoring wells (measurements made within one week of one another). Each point represents a monitoring well water level measurement (y-axis) and a driller reported static water level measurement (x-axis) for two wells that are located close together, at similar depths, have similar land surface elevations at their tops, and have measurements made within seven days of one another (but more than one day apart). We outline each point in light blue so that the density of partially-overlapping points can be interpreted more easily.  top panel presents all points with water levels within 350 m of the land surface (an interval including nearly every point in the dataset); the lower panel presents all points with water levels within 50m of the land surface (i.e., the lower plot represents a "zoom in" of the lower-left corner of the top panel).

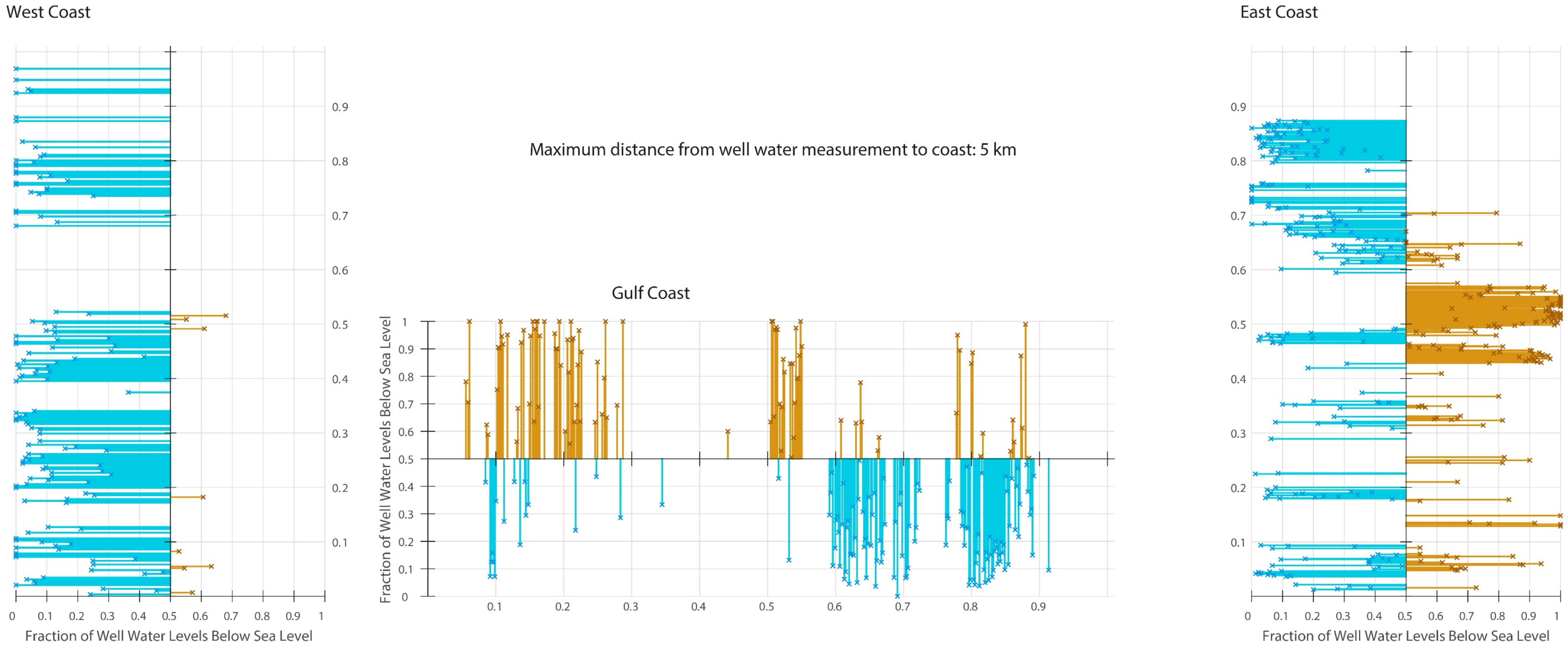


**Supplementary Figure 6.** Well water level elevations relative to sea level along contiguous U.S. coasts. The map in main text Figure 4 displays fractional distances along each coast corresponding to the y-axes (West and East Coasts) and x-axis (Gulf Coast). Colors along the coastlines represent 20 km segments of coast; orange segments represent those where at least half of the measured well water levels are below sea level and blue segments are those where more than half of well water levels are above sea level. Our analysis was completed using well water level measurements made within 5 km of a coastline (note: results presented in the main text were calculated using well water level measurements made within 10 km of the nearest coast). The three bar plots present the fraction of well water level measurements that are below sea level; each bar represents one 20 km coastline segment. We only display results for coastline segments with at least 10 well water level measurements made within 5 km of the coast.

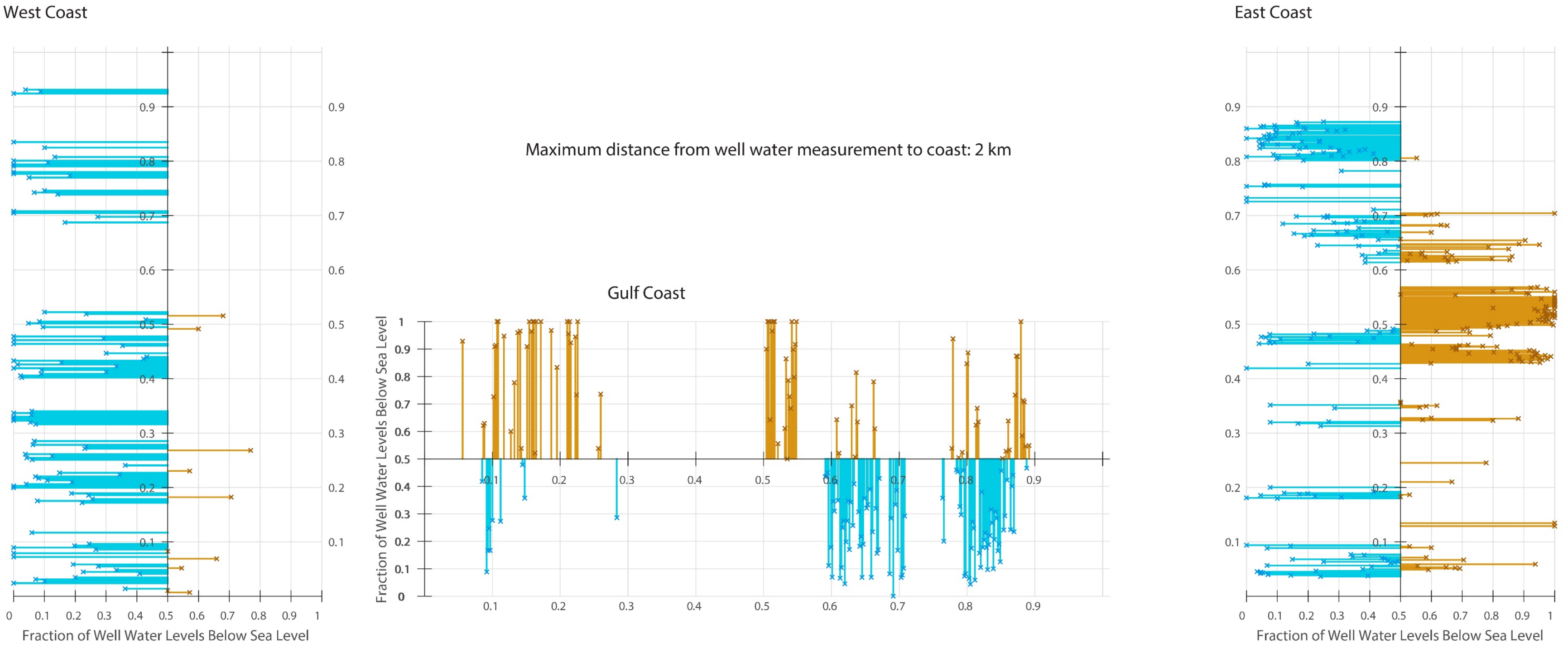


**Supplementary Figure 7.** Well water level elevations relative to sea level along contiguous U.S. coasts. The map in main text Figure 4 displays fractional distances along each coast corresponding to the y-axes (West and East Coasts) and x-axis (Gulf Coast). Colors along the coastlines represent 20 km segments of coast; orange segments represent those where at least half of the measured well water levels are below sea level and blue segments are those where more than half of well water levels are above sea level. Our analysis was completed using well water level measurements made within 2 km of a coastline (note: results presented in the main text were calculated using well water level measurements made within 10 km of the nearest coast). The three bar plots present the fraction of well water level measurements that are below sea level; each bar represents one 20 km coastline segment. We only display results for coastline segments with at least 10 well water level measurements made within 2 km of the coast.

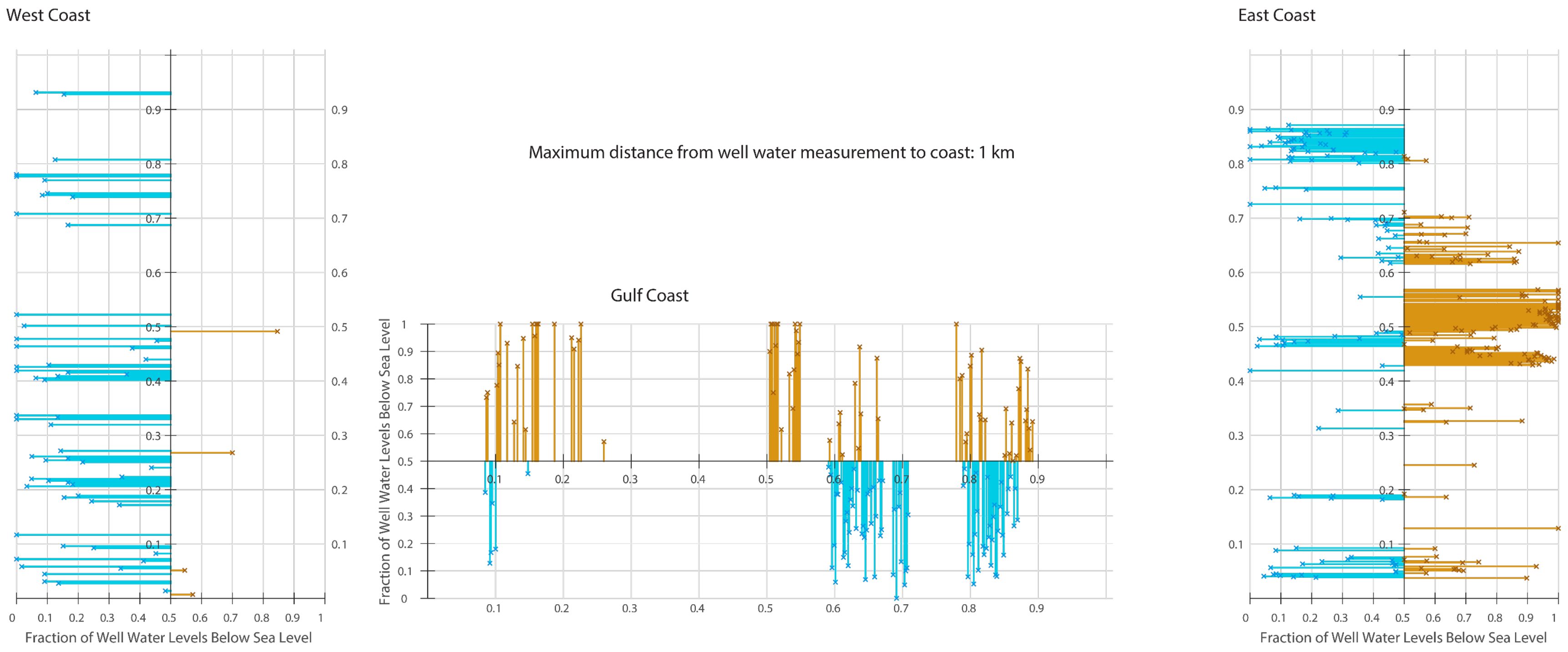


**Supplementary Figure 8.** Well water level elevations relative to sea level along contiguous U.S. coasts. The map in main text Figure 4 displays fractional distances along each coast corresponding to the y-axes (West and East Coasts) and x-axis (Gulf Coast). Colors along the coastlines represent 20 km segments of coast; orange segments represent those where at least half of the measured well water levels are below sea level and blue segments are those where more than half of well water levels are above sea level. Our analysis was completed using well water level measurements made within 1 km of a coastline (note: results presented in the main text were calculated using well water level measurements made within 10 km of the nearest coast). The three bar plots present the fraction of well water level measurements that are below sea level; each bar represents one 20 km coastline segment. We only display results for coastline segments with at least 10 well water level measurements made within 1 km of the coast.

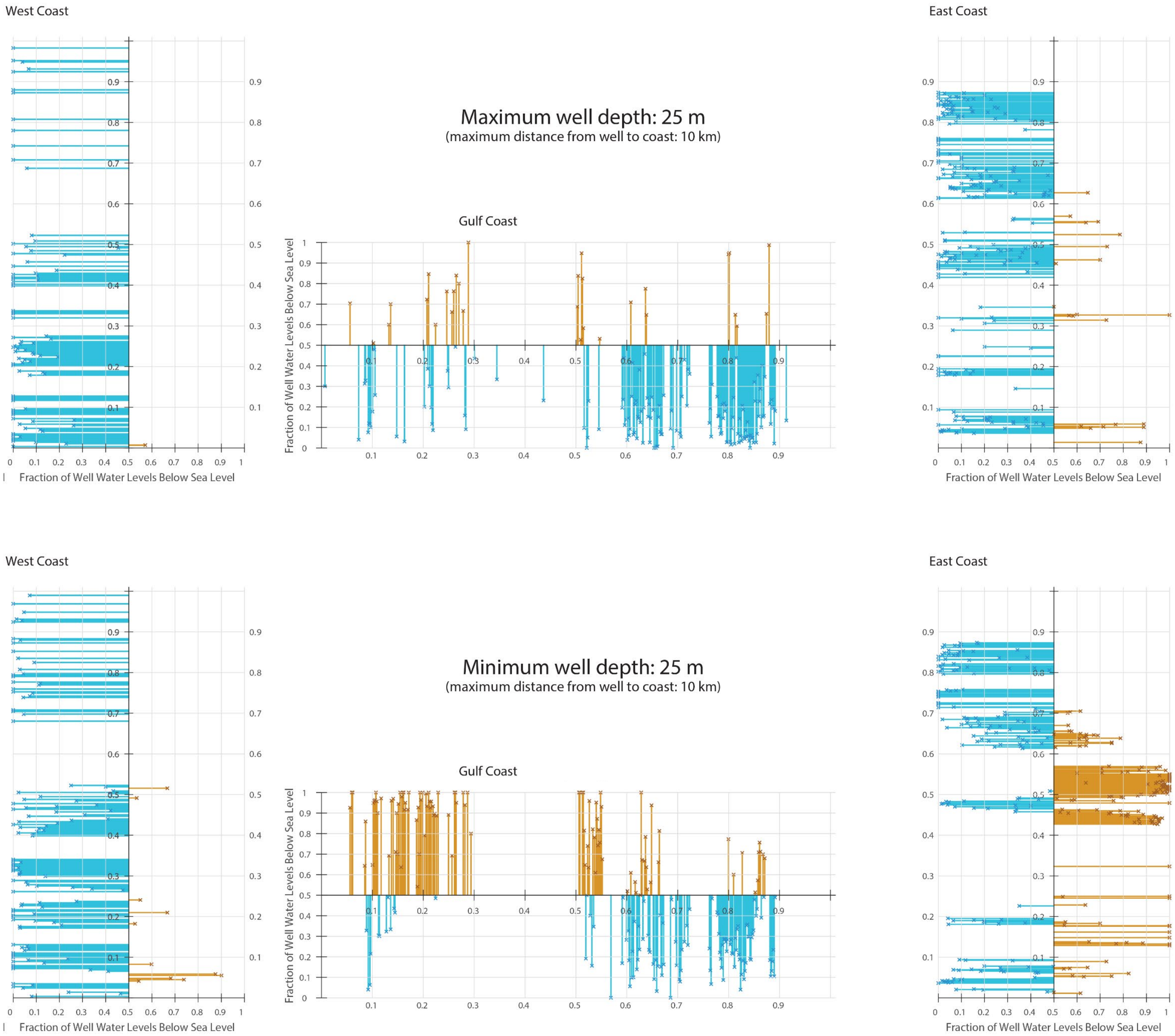


**Supplementary Figure 9.** Well water level elevations relative to sea level along contiguous U.S. coasts using wells shallower than 25m (top) or deeper than 25m (bottom). The display mimics main text Figure 4 (i.e., fractional distances along each coast corresponding to the y-axes (West and East Coasts) and x-axis (Gulf Coast)). Colors along the coastlines represent 20 km segments of coast; orange segments represent those where at least half of the measured well water levels are below sea level and blue segments are those where more than half of well water levels are above sea level. Our analysis was completed using well water level measurements made within 10 km of a coastline. Each bar represents one 20 km coastline segment. We only display results for coastline segments with at least 10 well water level measurements made within 10 km of the coast.

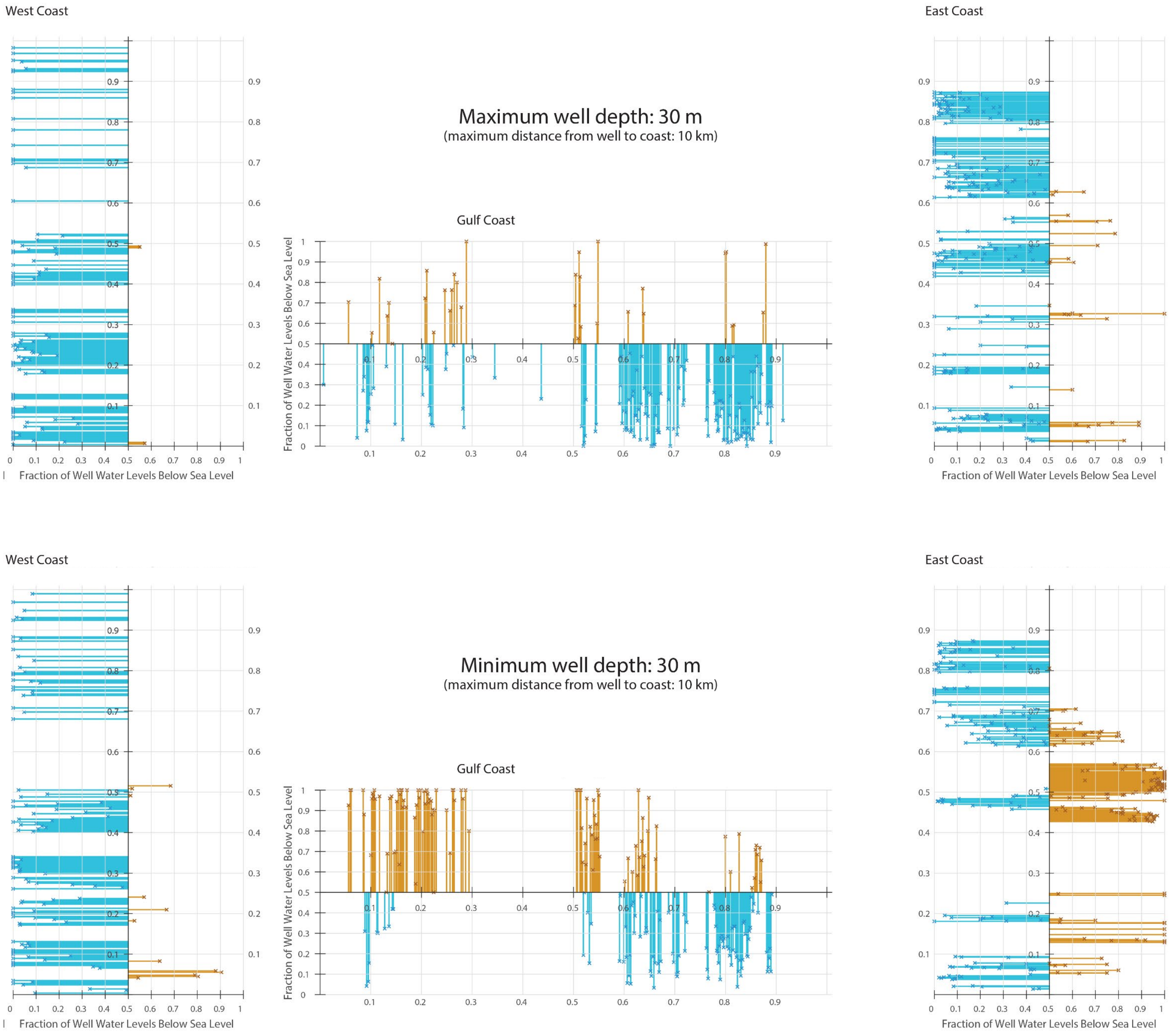


**Supplementary Figure 10.** Well water level elevations relative to sea level along contiguous U.S. coasts using wells shallower than 30m (top) or deeper than 30m (bottom). The display mimics main text Figure 4 (i.e., fractional distances along each coast corresponding to the y-axes (West and East Coasts) and x-axis (Gulf Coast)). Colors along the coastlines represent 20 km segments of coast; orange segments represent those where at least half of the measured well water levels are below sea level and blue segments are those where more than half of well water levels are above sea level. Our analysis was completed using well water level measurements made within 10 km of a coastline. Each bar represents one 20 km coastline segment. We only display results for coastline segments with at least 10 well water level measurements made within 10 km of the coast.

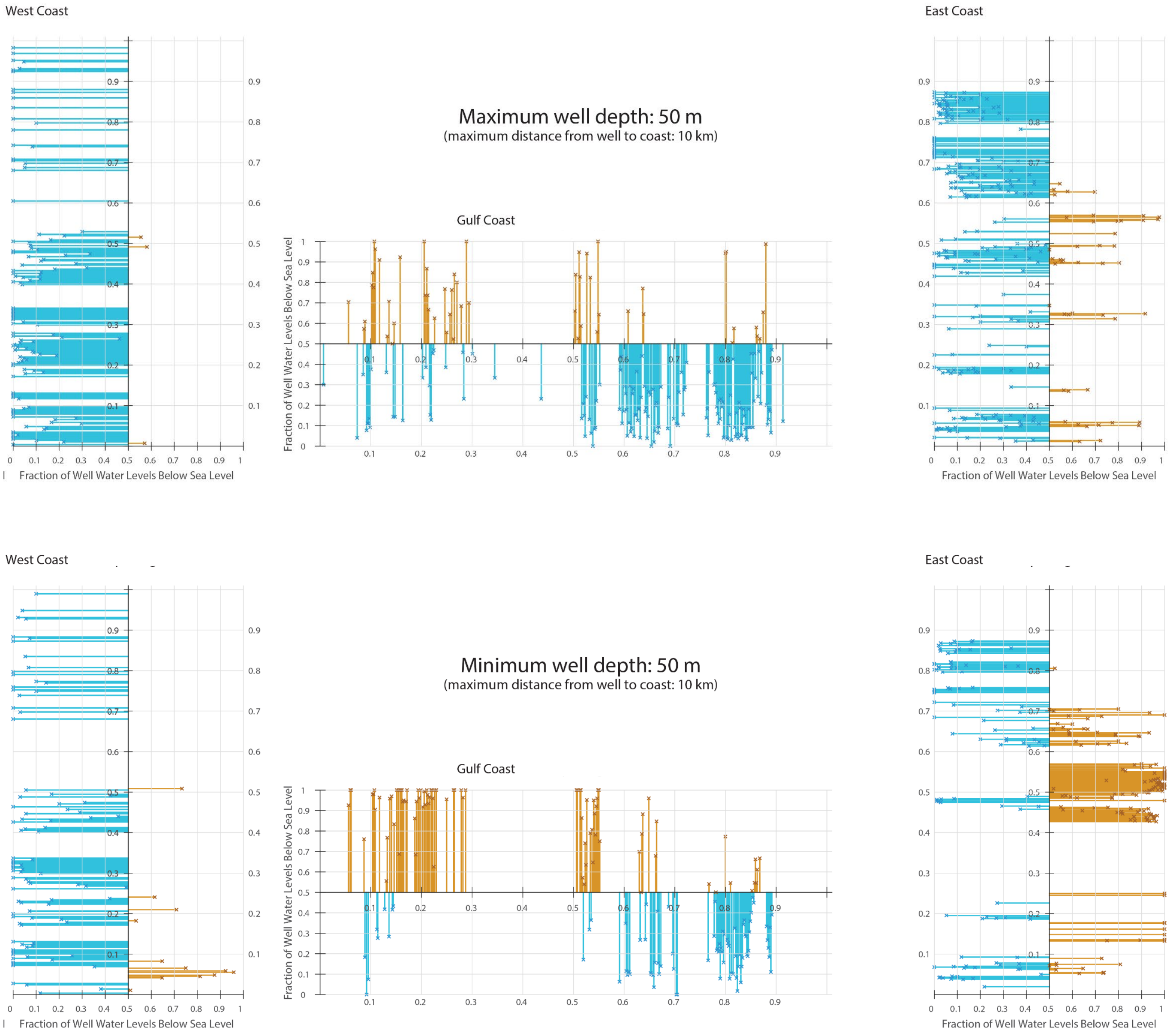


**Supplementary Figure 11.** Well water level elevations relative to sea level along contiguous U.S. coasts using wells shallower than 50m (top) or deeper than 50m (bottom). The display mimics main text Figure 4 (i.e., fractional distances along each coast corresponding to the y-axes (West and East Coasts) and x-axis (Gulf Coast)). Colors along the coastlines represent 20 km segments of coast; orange segments represent those where at least half of the measured well water levels are below sea level and blue segments are those where more than half of well water levels are above sea level. Our analysis was completed using well water level measurements made within 10 km of a coastline. Each bar represents one 20 km coastline segment. We only display results for coastline segments with at least 10 well water level measurements made within 10 km of the coast.

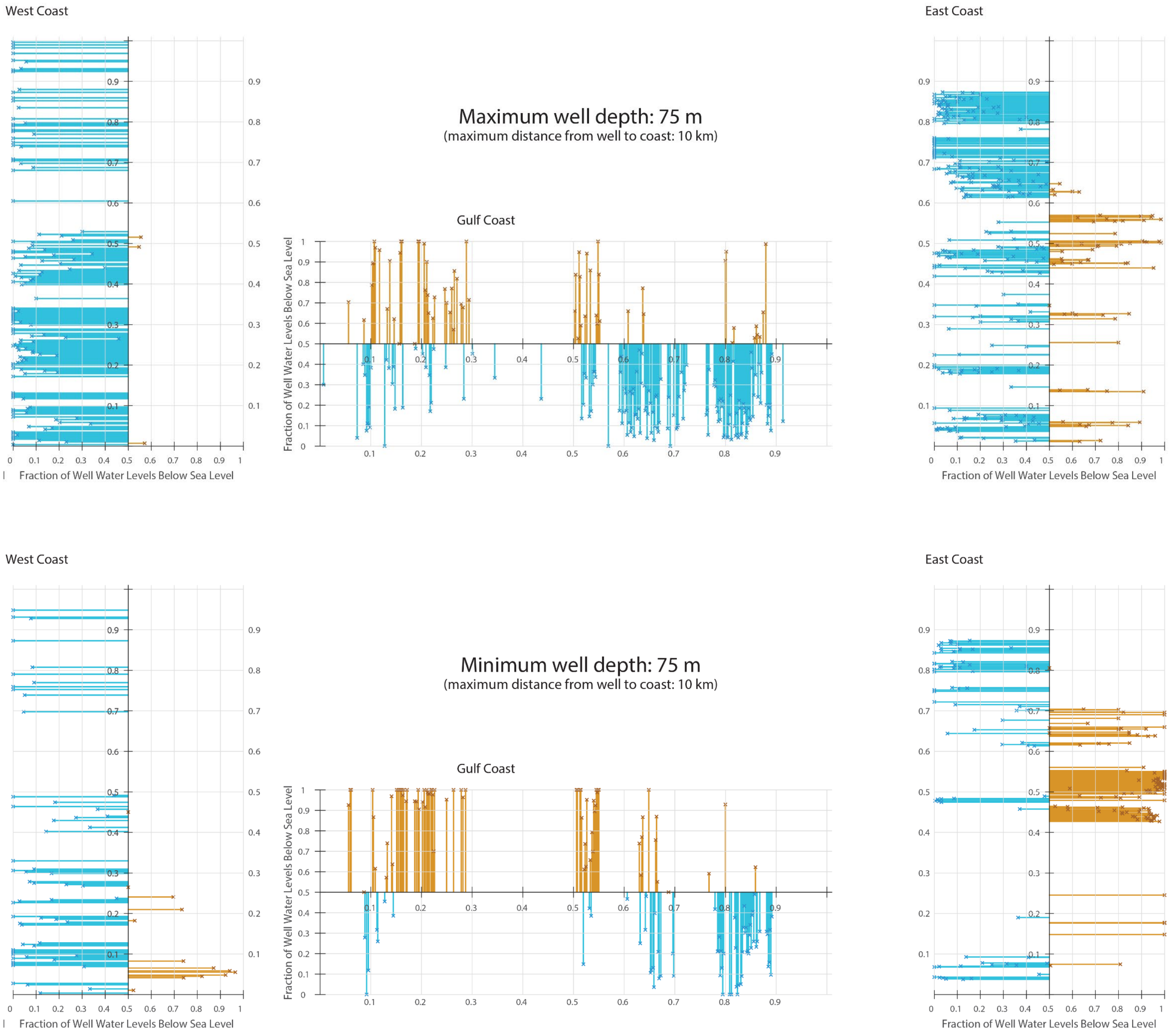


**Supplementary Figure 12.** Well water level elevations relative to sea level along contiguous U.S. coasts using wells shallower than 75m (top) or deeper than 75m (bottom). The display mimics main text Figure 4 (i.e., fractional distances along each coast corresponding to the y-axes (West and East Coasts) and x-axis (Gulf Coast)). Colors along the coastlines represent 20 km segments of coast; orange segments represent those where at least half of the measured well water levels are below sea level and blue segments are those where more than half of well water levels are above sea level. Our analysis was completed using well water level measurements made within 10 km of a coastline. Each bar represents one 20 km coastline segment. We only display results for coastline segments with at least 10 well water level measurements made within 10 km of the coast.

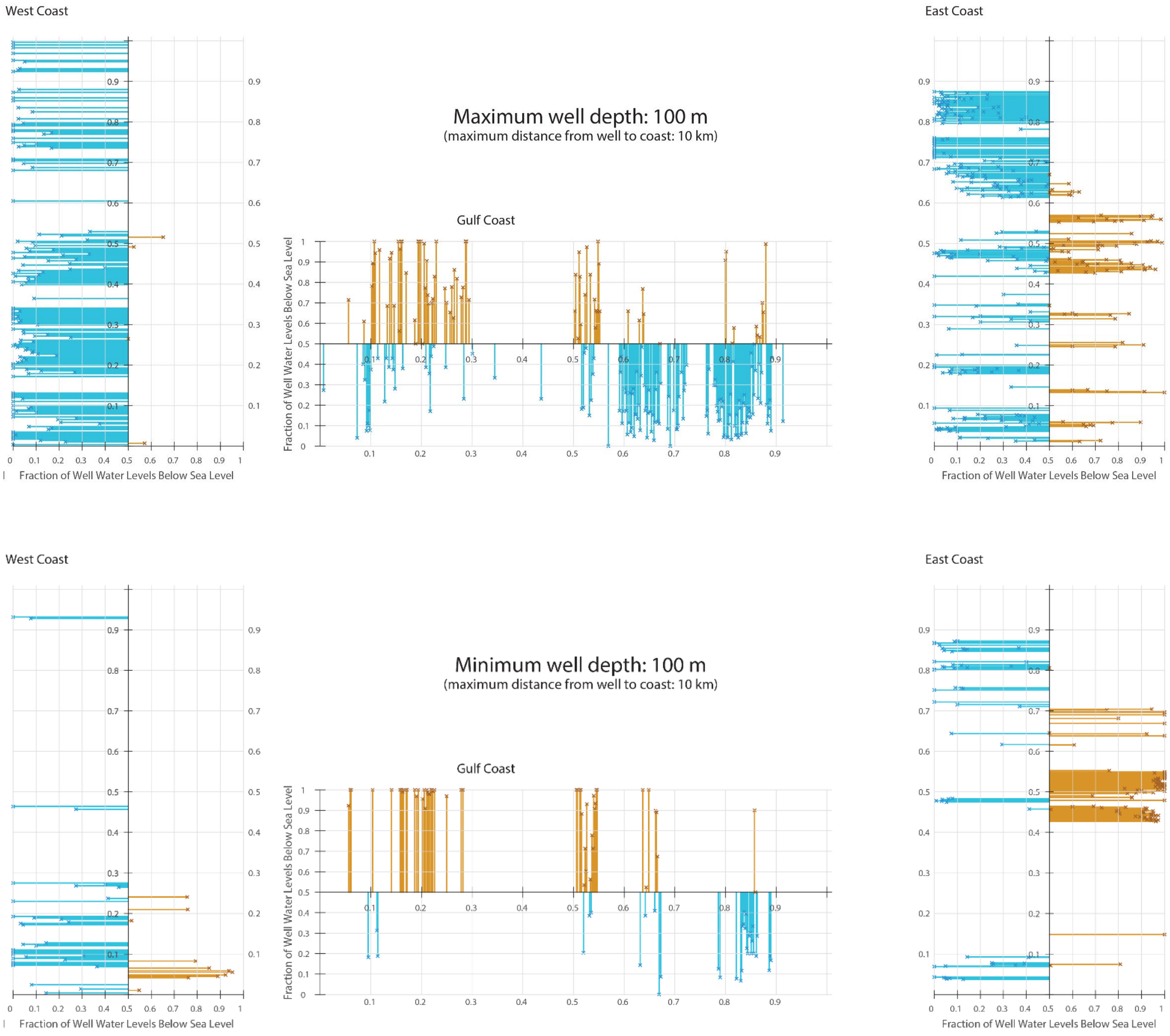


**Supplementary Figure 13.** Well water level elevations relative to sea level along contiguous U.S. coasts using wells shallower than 100 (top) or deeper than 100m (bottom). The display mimics main text Figure 4 (i.e., fractional distances along each coast corresponding to the y-axes (West and East Coasts) and x-axis (Gulf Coast)). Colors along the coastlines represent 20 km segments of coast; orange segments represent those where at least half of the measured well water levels are below sea level and blue segments are those where more than half of well water levels are above sea level. Our analysis was completed using well water level measurements made within 10 km of a coastline. Each bar represents one 20 km coastline segment. We only display results for coastline segments with at least 10 well water level measurements made within 10 km of the coast.

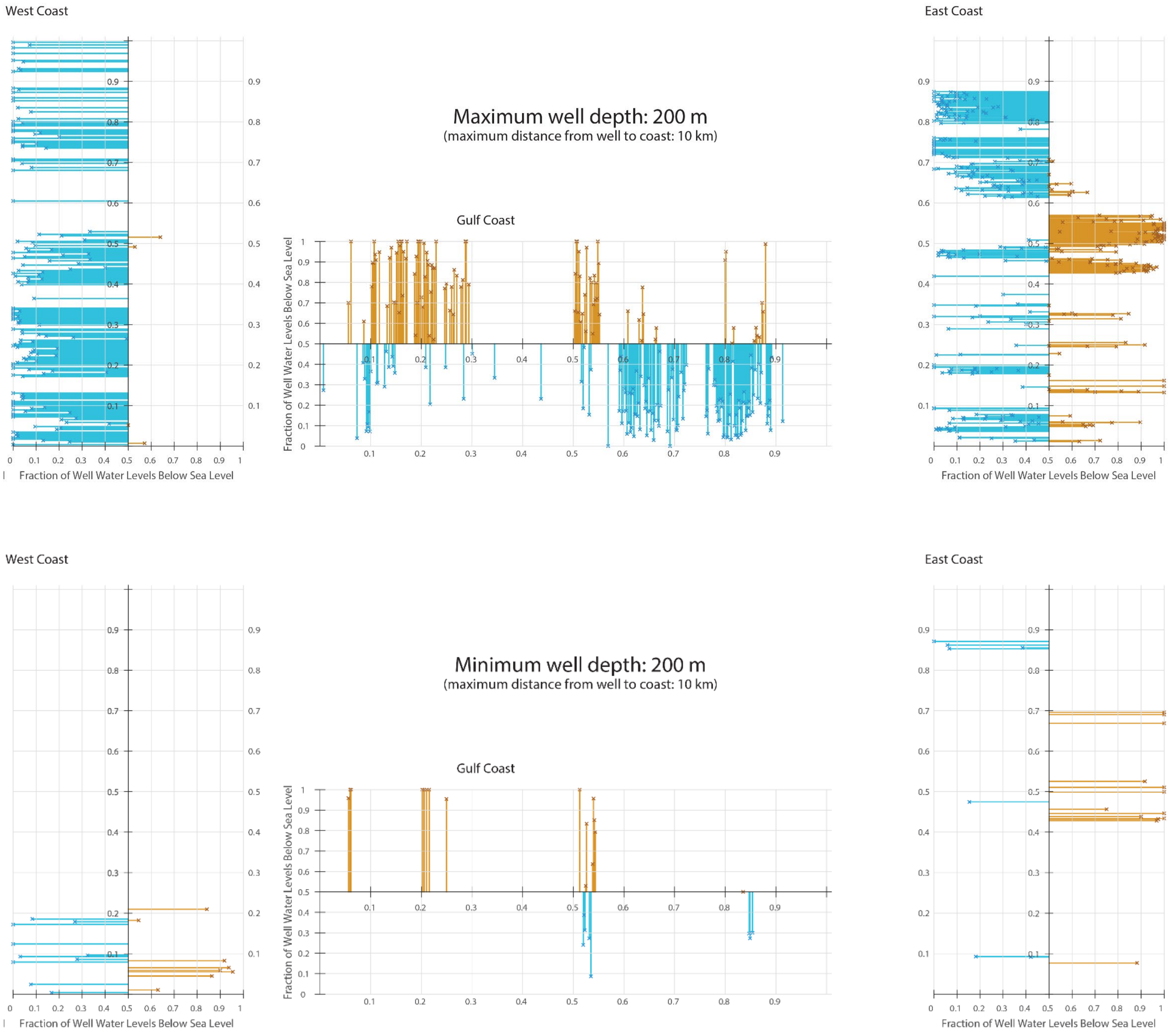


**Supplementary Figure 14.** Well water level elevations relative to sea level along contiguous U.S. coasts using wells shallower than 200m (top) or deeper than 200m (bottom). The display mimics main text Figure 4 (i.e., fractional distances along each coast corresponding to the y-axes (West and East Coasts) and x-axis (Gulf Coast)). Colors along the coastlines represent 20 km segments of coast; orange segments represent those where at least half of the measured well water levels are below sea level and blue segments are those where more than half of well water levels are above sea level. Our analysis was completed using well water level measurements made within 10 km of a coastline. Each bar represents one 20 km coastline segment. We only display results for coastline segments with at least 10 well water level measurements made within 10 km of the coast

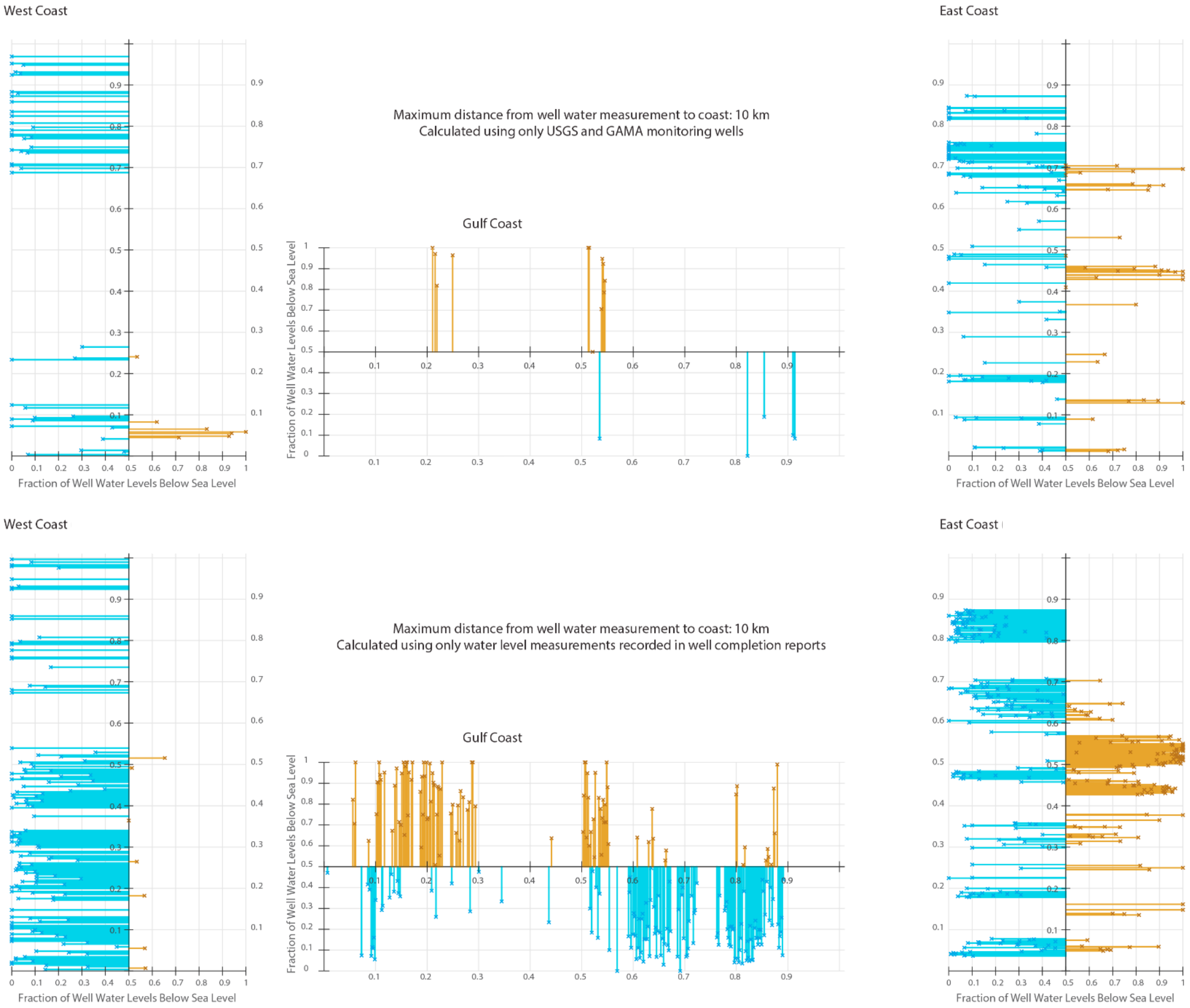


**Supplementary Figure 15.** Comparison of fraction of well water levels that lie below sea level along contiguous USA coasts calculated using only monitoring wells (upper panels) versus only driller report water levels (lower panels). Orange and blue segments represent those where more than half of the measured well levels are below and above sea level, respectively. Each bar represents one 20-km segment, reaching 10 km inland. The numbers on the axes (x-axis for Gulf, y-axis for West and East) indicate the fractional distance along the coastline from south to north or west to west. We only plot a bar for a 20 km-long segment if the analyzed dataset contains at least ten unique wells with at least one post-2000 water level.

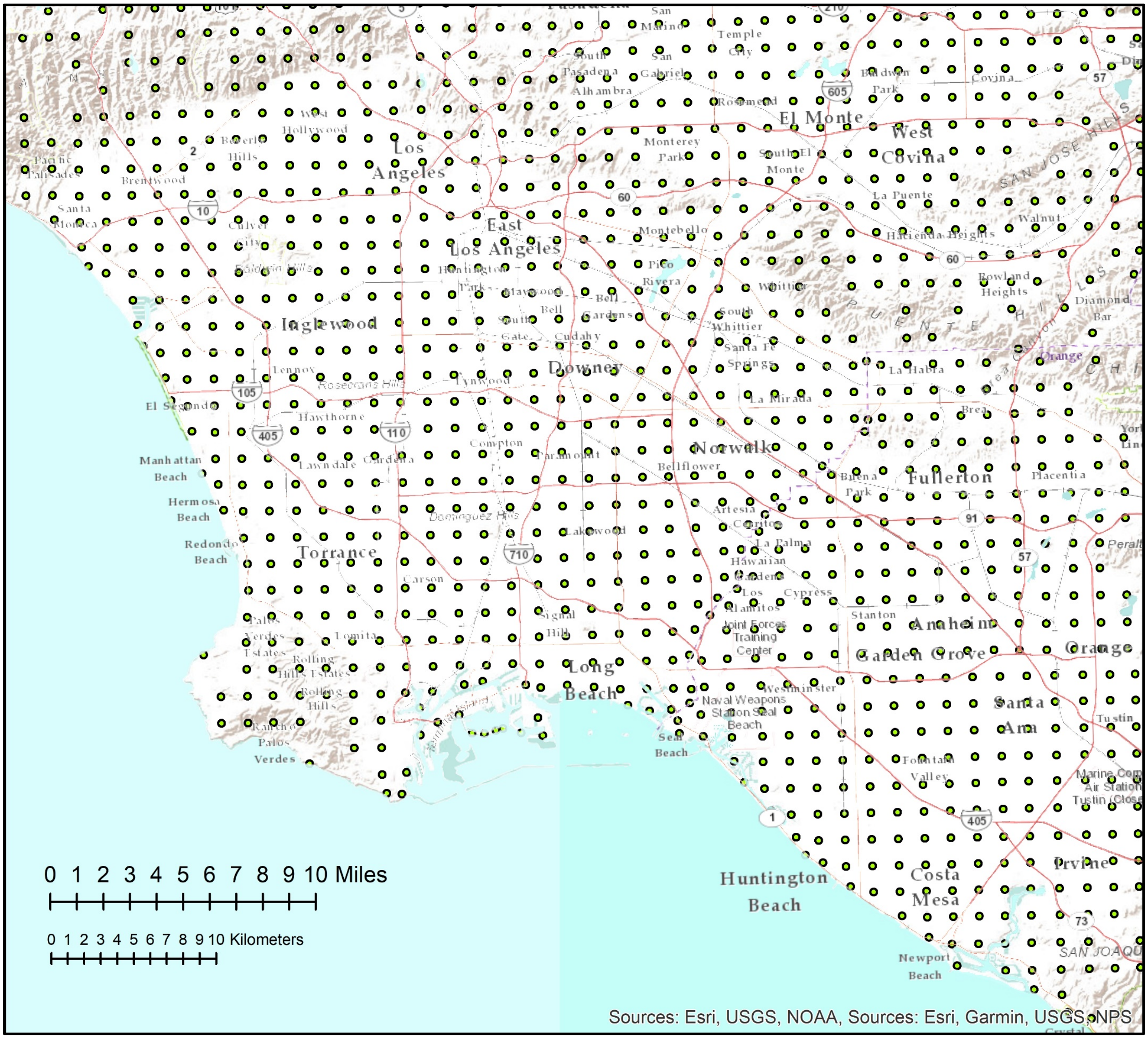


**Supplementary Figure 16.** Map highlighting how constructed well locations are geocoded to the nearest ±1 mile. The map displays the Los Angeles Basin; most (but not all) constructed wells are geocoded to centroids of township-range-section quadrants (green circles display well locations). Map created in ArcGIS© 10.5.1. Basemap data from https://www.arcgis.com/home/item.html?id=fe44cf9a739848939988addfeba473e4 (accessed May 12, 2020).

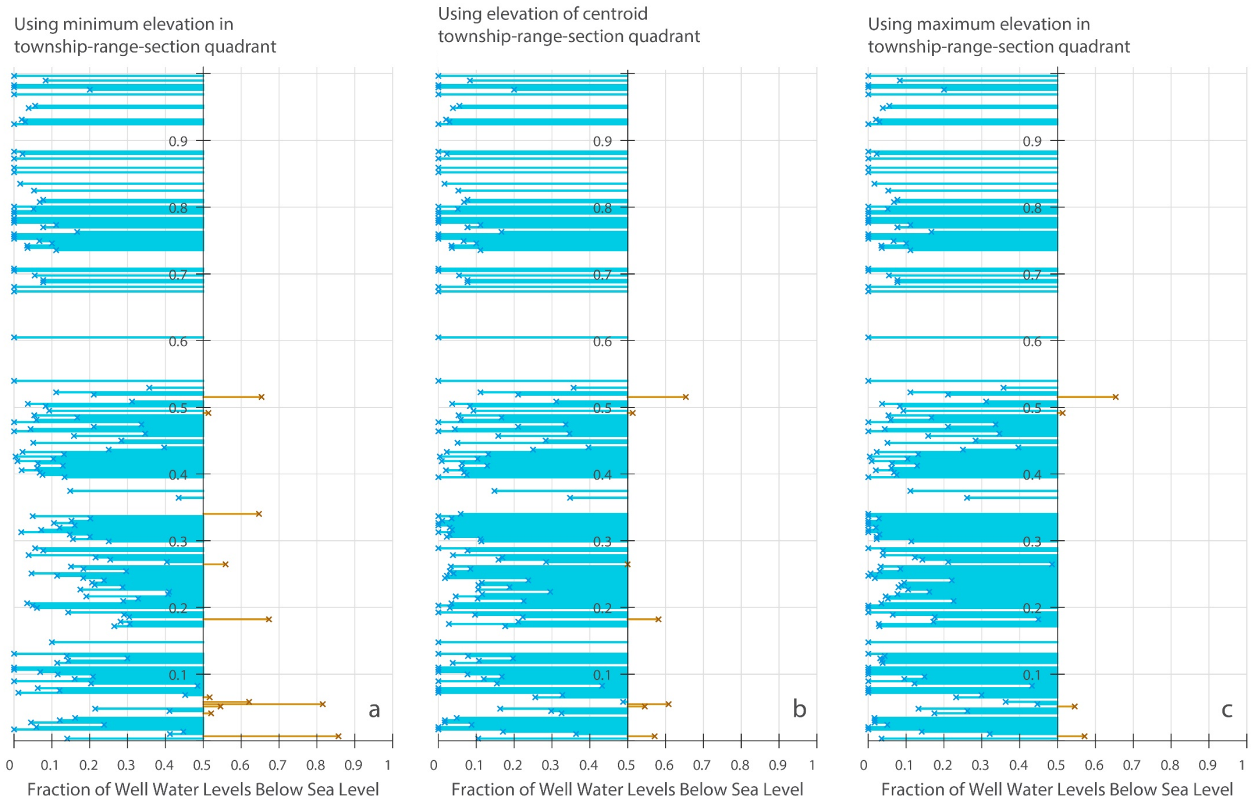


**Supplementary Figure 17.** West Coast well water level elevations relative to sea level under three sensitivity analyses. The three bar plots present the fraction of well water level measurements that are below sea level. Each bar represents a 20 km segment of coast; orange bars represent those where at least half of the measured well water levels are below sea level and blue bars are those where more than half of well water levels are above sea level. Panel (a) presents results for the assumption that wellhead elevations are the minimum elevation within the wellhead location uncertainty (i.e., the minimum elevation within the township-range-section among wells geocoded to the township-range-section centroid). Panel (b) presents results derived using the elevation of the centroid of township-range-sections. Panel (c) presents results for the assumption that wellhead elevations are the maximum elevation within the wellhead location uncertainty (i.e., the maximum elevation within the township-range-section among wells geocoded to the township-range-section centroid). We only display results for coastline segments with at least 10 well water level measurements made within 10 km of the coast. The locations of fractional distances along the West Coast (i.e., the y-axis scale) are displayed in Figure 4 of the main text.

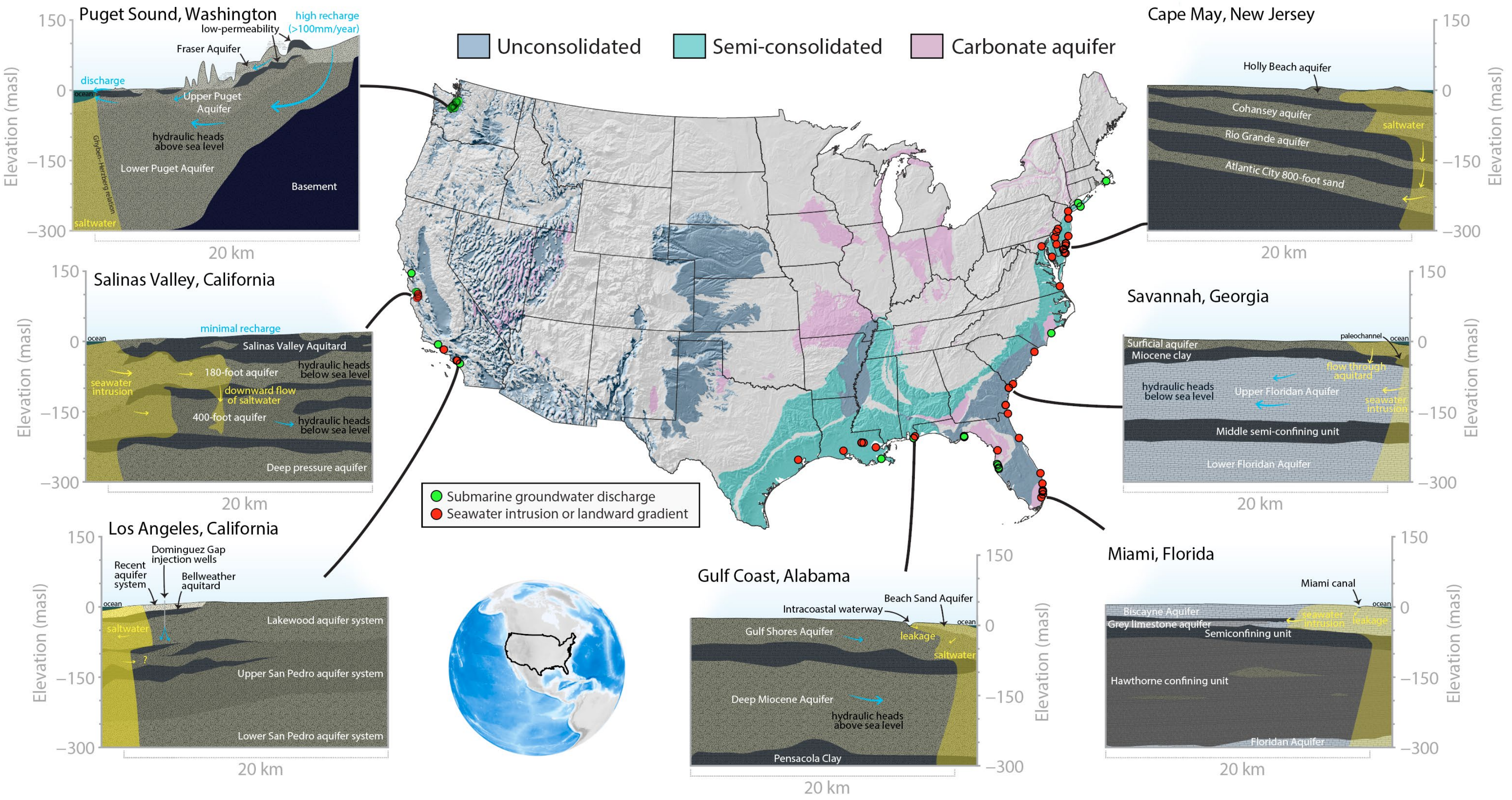

Unconsolidated
Semi-consolidated
Carbonate aquifer
Submarine groundwater discharge
Seawater intrusion or landward gradient
Puget Sound, Washington
high recharge (>100mm/year)
low-permeability
Fraser Aquifer
discharge
ocean
Upper Puget Aquifer
hydraulic heads above sea level
Lower Puget Aquifer
Basement
Ghyben-Herzberg relation
saltwater
Elevation (masl)
150
0
−150
−300
20 km
Salinas Valley, California
minimal recharge
Salinas Valley Aquitard
180-foot aquifer
hydraulic heads below sea level
seawater intrusion
downward flow of saltwater
400-foot aquifer
Deep pressure aquifer
Los Angeles, California
Dominguez Gap injection wells
Recent aquifer system
Bellweather aquitard
Lakewood aquifer system
Upper San Pedro aquifer system
Lower San Pedro aquifer system
Cape May, New Jersey
Holly Beach aquifer
Cohansey aquifer
Rio Grande aquifer
Atlantic City 800-foot sand
Savannah, Georgia
Surficial aquifer
Miocene clay
paleochannel
flow through aquitard
hydraulic heads below sea level
Upper Floridan Aquifer
seawater intrusion
Middle semi-confining unit
Lower Floridan Aquifer
Gulf Coast, Alabama
Beach Sand Aquifer
Intracoastal waterway
Gulf Shores Aquifer
leakage
Deep Miocene Aquifer
hydraulic heads above sea level
Pensacola Clay
Miami, Florida
Miami canal
Biscayne Aquifer
Grey limestone aquifer
Semiconfining unit
Hawthorne confining unit
Floridan Aquifer

**Supplementary Figure 18 (previous page).** Compilation of local studies of submarine groundwater discharges and seawater intrusion. These local-scale studies use a variety of approaches, including hydrological models, seepage meters, and solute concentration measurements (see review by Santos, I. R., Eyre, B. D., Huettel, M. The driving forces of porewater and groundwater flow in permeable coastal sediments: A review. Estuar. Coast. Shelf Sci. 98, 1–15 (2012)). We show locations where local-scale studies have demonstrated or simulated the occurrence of submarine groundwater discharge (blue points), or a landward gradient or seawater intrusion (red points). Submarine groundwater discharge implies a seaward hydraulic gradient; conversely, seawater intrusion suggests that a landward hydraulic gradient may exist (though some seawater intrusion can occur even where well water levels lie above sea level – the "Ghijben-Herzberg" principle). For primary results and discussion of important local considerations see (i) McCoy, C. A., Corbett, D. R. Review of submarine groundwater discharge (SGD) in coastal zones of the Southeast and Gulf Coast regions of the United States with management implications. J. Environ. Manage. 90, 644–651 (2009); (ii) Sawyer, A. H., Michael, H. A., Schroth, A. W. From soil to sea: the role of groundwater in coastal critical zone processes. Wiley Interdiscip. Rev. Water 3, 706–726 (2016). and (iii) Lecher, A., Mackey, K. Synthesizing the effects of submarine groundwater discharge on marine biota. Hydrology 5, 60 (2018). For reviews of studies of submarine groundwater discharge research and seawater intrusion prior to the year 2000 see refs. (i) Taniguchi, M., Burnett, W. C., Cable, J. E., Turner, J. V. Investigation of submarine groundwater discharge. Hydrological Processes, 16, 2115–2129 (2002) and (ii) Barlow, P. M., Reichard, E. G. Saltwater intrusion in coastal regions of North America. Hydrogeol. J. 18, 247–260 (2010). For primary references see Note 7 (Supplementary Table 5), which reviews 108 studies. Although our analysis focuses on identifying areas with landward hydraulic gradients, we also compile studies reporting submarine groundwater discharges to help distinguish unstudied areas from those with known seaward hydraulic gradients. We also present a series of conceptual models based on previous studies as cross sections. In Puget Sound[12-14] (Washington state in top-left panel), high relief and recharge rates and modest groundwater use create seaward hydraulic gradients and concomitant submarine groundwater discharge. In the Salinas Valley[15-17] (California), decades of high groundwater pumping and limited local recharge (due to a shallow aquitard) have increased landward hydraulic gradients and induced seawater intrusion. Seawater has moved several kilometers inland in both the shallow ("180-foot") aquifer and the deeper ("400-foot") aquifer; intruded seawater is likely to be also seeping downward from the 180-foot aquifer into the 400-foot aquifer. In the West Coast Basin[18,19] (Los Angeles, California), high groundwater withdrawals have created landward hydraulic gradients and seawater intrusion, but the installation and operation of injection wells at "Dominguez Gap" have increased hydraulic heads in the aquifer close to the coast and have likely helped slow seawater intrusion. In the Gulf Coast of Alabama[20], hydraulic heads lie just above sea level yet some intrusion has occurred. Leakage from the Intracoastal Waterway may also be salinizing parts of the shallow unconfined aquifer. Near Miami[39,40] (Florida), a shallow carbonate aquifer ("Biscayne") has experienced seawater intrusion, likely exacerbated by the construction of leaky canals. In Savannah[27] (Georgia), groundwater withdrawals have drawn down a piezometric surface that once lay above sea level to now lie below sea level, creating a landward hydraulic gradient. In Cape May[35] (New Jersey), landward gradients are clearly reflected in piezometric data for confined aquifers (e.g., the "Cohansey" and "Atlantic City 800-foot sand"), highlighting the potential vulnerability of confined aquifers to seawater intrusion, should a pathway exist for seawater to migrate into and through these aquifers.

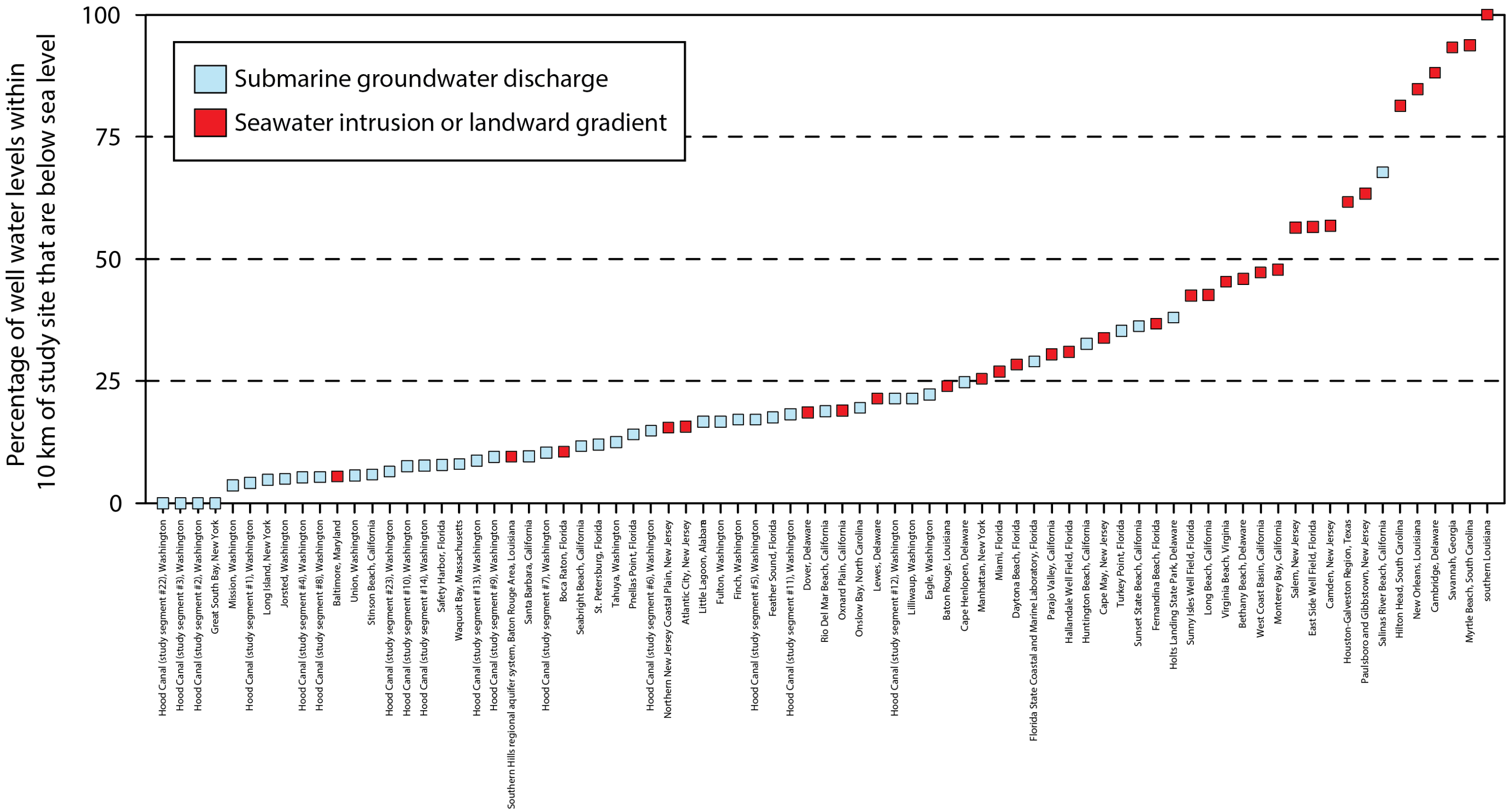


**Supplementary Figure 19.** Comparison of the well water levels presented in this study (i.e., Figure 2 main text) at locations where previous studies have identified seawater intrusion or submarine groundwater discharges (i.e., locations in Figure 1 main text and Supplementary Table 5). Each square represents one location where previous studies have identified seawater intrusion (or a landward gradient) or submarine groundwater discharges (i.e., a location from Supplementary Table 5). The color of the square signifies the findings of previous research, namely whether submarine groundwater discharge was confirmed (blue squares) or if seawater intrusion or a landward hydraulic gradient was confirmed (red squares). The position of each point in the vertical dimension corresponds to the percentage of well water level observations from our study (i.e., those in Figure 2) within 10 km of the study location that have an elevation below sea level. Locations where previous research has identified a landward hydraulic gradient or seawater intrusion (i.e., red squares) are confirmed to often have nearby well water level elevations that lie below sea level; that is, red squares represent local-scale study locations where a landward gradient (or seawater intrusion) has been identified, and these locations are surrounded by well water level measurements that are frequently below sea level. We only plot locations with at least n=10 well water level measurements within 10 km of the study location.

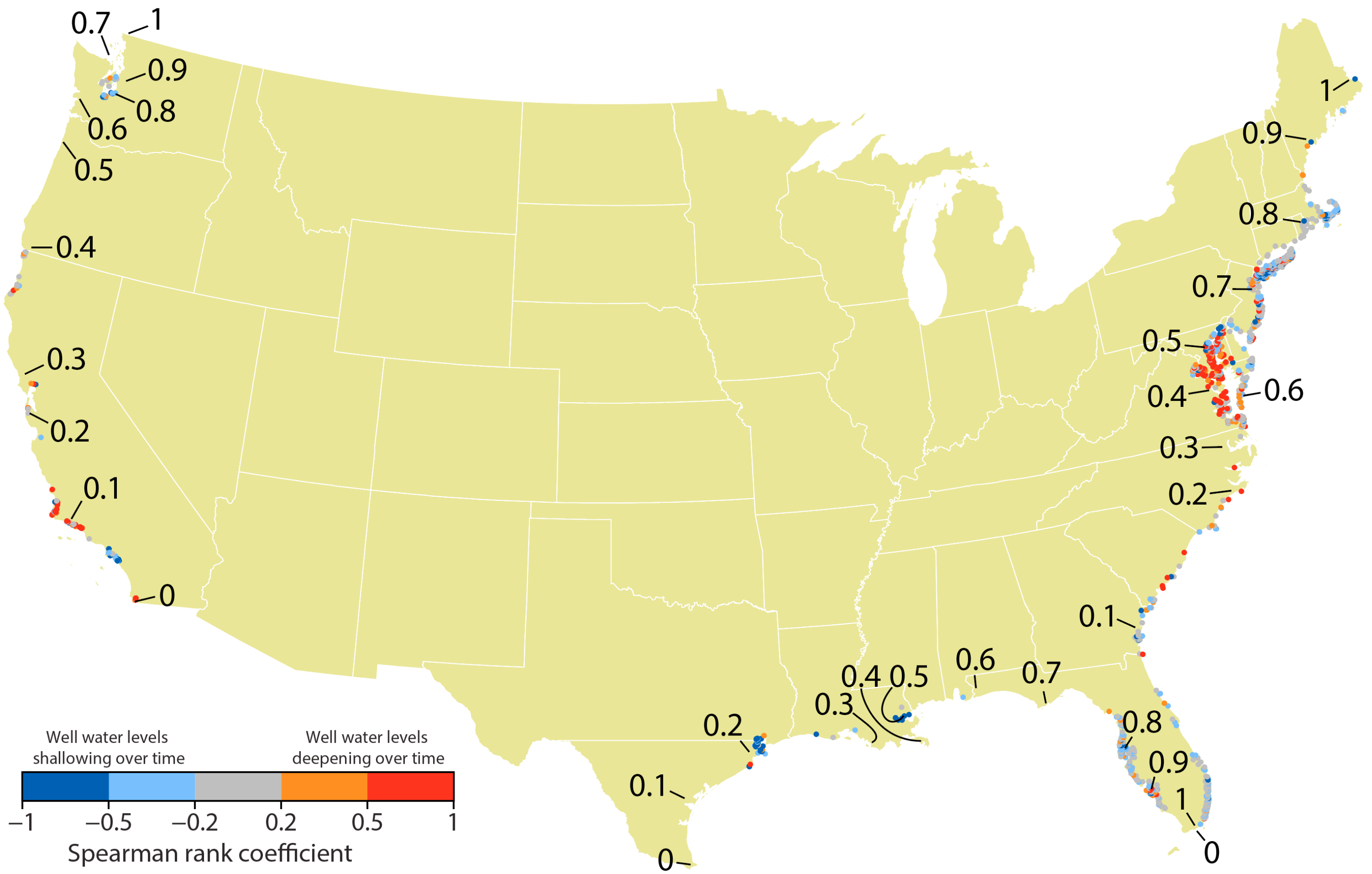


**Supplementary Figure 20.** Well water level variations over time from 2000-2015. The numbers on the map indicate the fractional distance along the coastline from south to north or west to east (consistent with fractional distances presented in main text Figure 4). Each point represents one monitoring well that met our criteria for analyses (text in Note 9). The color of each point corresponds to the Spearman rank correlation coefficient of [depth to water in well] versus [measurement date]. Positive coefficients (i.e., orange and red) are consistent with a decline in well water levels over time (i.e., an increase in the rank of [depth below land surface to water] as the rank of [measurement date] increases—consistent with a deepening of well water levels over time); conversely, negative coefficients (i.e., blue) are consistent with increases in well water levels over time (i.e., decrease in the rank of [depth below land surface to water] as the rank of [measurement date] increases – consistent with a shallowing of well water levels over time.

## (C) Supplementary Tables

**Supplementary Table 1.** Sensitivity of our results to the maximum distance from wells to the coast considered in results

| | **20 km coastal segments where at least half of all well water level measurements lie below sea level** | | |
|---|---|---|---|
| **Maximum distance** | **West Coast** | **Gulf Coast** | **East Coast** |
| 10 km ** (Figure 4 main text) | n=7 of n=166 (4.2% of studied segments) | n=102 of n=250 (40.8% of studied segments) | n=163 of n=420 (38.8% of studied segments) |
| 5 km *** (Supplementary Figure 6) | n=8 of n=138 (5.8% of studied segments) | n=96 of n=225 (42.7% of studied segments) | n=161 of n=379 (42.5% of studied segments) |
| 2 km *** (Supplementary Figure 7) | n=10 of n=103 (9.7% of studied segments) | n=83 of n=183 (45.3% of studied segments) | n=149 of n=304 (49.0% of studied segments) |
| 1 km *** (Supplementary Figure 8) | n=4 of n=62 (6.5% of studied segments) | n=75 of n=159 (47.2% of studied segments) | n=146 of n=257 (56.8% of studied segments) |

* only coastline segments where at least n=10 well water level elevation measurements are available were analyzed (i.e., the number of studied segments decreases as the maximum distance decreases)

** see Figure 3 main text

*** see Supplementary Figures 6, 7 and 8

**Supplementary Table 2.** Fractions of analyzed coastlines where the majority of well water levels lie below sea level

| Supplementary figure | Well depth interval (m) | **West Coast **** segments with majority of well water levels lie below sea level | | **Gulf Coast **** segments with majority of well water levels lie below sea level | | **East Coast **** segments with majority of well water levels lie below sea level | |
|---|---|---|---|---|---|---|---|
| **Supp. Fig. 9** | **0 – 25 m** | 1/88 | 1.1% | 30/178 | 16.9% | 21/248 | 8.5% |
| | **>25 m** | 11/131 | 8.4% | 102/216 | 47.2% | 149/311 | 47.9% |
| **Supp. Fig. 10** | **0 – 30 m *** | 2/101 | 2.0% | 33/187 | 17.6% | 29/264 | 11.0% |
| | **>30 m *** | 12/125 | 9.6% | 103/209 | 49.3% | 149/295 | 50.5% |
| **Supp. Fig. 11** | **0 – 50 m** | 3/125 | 2.4% | 53/207 | 25.6% | 46/292 | 15.8% |
| | **>50 m** | 12/105 | 11.4% | 87/169 | 51.5% | 148/245 | 60.4% |
| **Supp. Fig. 12** | **0 – 75 m** | 3/140 | 2.1% | 63/218 | 28.9% | 62/315 | 19.7% |
| | **>75 m** | 13/72 | 18.1% | 72/139 | 51.8% | 119/192 | 62.0% |
| **Supp. Fig. 13** | **0 – 100 m** | 4/146 | 2.7% | 76/228 | 33.3% | 87/330 | 26.4% |
| | **>100 m** | 11/45 | 24.4% | 54/88 | 61.4% | 98/152 | 64.5% |
| **Supp. Fig. 14** | **0 – 200 m** | 4/155 | 2.6% | 99/244 | 40.6% | 144/381 | 37.8% |
| | **>200 m** | 8/18 | 44.4% | 16/24 | 66.7% | 14/21 | 66.7% |

* this 30 m threshold (Supplementary Figure 10) is shown for consistency with the vertical scale studied by Sawyer, A. H., David, C. H., & Famiglietti, J. S. Continental patterns of submarine groundwater discharge reveal coastal vulnerabilities. Science, 353, 705-707 (2016).

**results presents as *x* / *y* (e.g., 1/88) can be interpreted as follows: "Among a total of *y* 20 km-long coastline segments meeting our criteria for analyses, the majority of well water levels lie below sea level in *x* of these segments"

**Supplementary Table 3.** Wells with post-2000 water level data close to the coast in monitoring well versus well completion report datasets

| | Number of unique wells with at least one post-2000 water level measurement | | |
|---|---|---|---|
| **Maximum distance **** | **West Coast** | **Gulf Coast** | **East Coast** |
| 10 km * | USGS/GAMA: 2,059<br>Driller report: 12,137 | USGS/GAMA: 706<br>Driller report: 141,426 | USGS/GAMA: 4,884<br>Driller report: 95,528 |
| 5 km | USGS/GAMA: 1,260<br>Driller report: 8,057 | Driller report: 99,054<br>USGS/GAMA: 425 | Driller report: 59,421<br>USGS/GAMA: 3,145 |
| 2 km | Driller report: 3,622<br>USGS/GAMA: 628 | Driller report: 53,975<br>USGS/GAMA: 209 | Driller report: 30,375<br>USGS/GAMA: 1,774 |
| 1 km | Driller report: 1,893<br>USGS/GAMA: 358 | Driller report: 30,515<br>USGS/GAMA: 107 | Driller report: 18,127<br>USGS/GAMA: 1,021 |

* sum of all values in this row (USGS/GAMA plus driller report) is 256,740 (the basis for the title of our paper: "Groundwater level observations in *250,000* coastal USA wells reveal scope of potential seawater intrusion")
** maximum distance represents the maximum distance from a well (with a post-2000 water level measurement) to the nearest coast.

**Supplementary Table 4.** Sensitivity of our results for the West Coast to the uncertainty in California wellhead locations

| | **20 km coastal segments where at least half of all well water level measurements lie below sea level** | | |
|---|---|---|---|
| **Maximum distance** | **Using lowest elevation in township-range-section quadrant** | **Using elevation of centroid of township-range-section quadrant **** | **Using highest elevation in township-range-section quadrant** |
| 10 km | n=11 of n=166 (6.6% of studied segments) | n=7 of n=166 (4.2% of studied segments) | n=4 of n=166 (2.4% of studied segments) |
| 5 km | n=14 of n=138 (10.1% of studied segments) | n=8 of n=138 (5.8% of studied segments) | n=6 of n=138 (4.3% of studied segments) |
| 2 km | n=20 of n=103 (19.4% of studied segments) * | n=10 of n=103 (9.7% of studied segments) | n=9 of n=103 (8.7% of studied segments) |

* maximum number of segments where at least half the observed well water levels lie below sea level. This value forms the basis of the following statement in the main text: *"Because well locations are particularly uncertain in California, we conducted sensitivity analyses for the West Coast and found that at least half of well water elevations could lie below sea level for as many as 6.9% of all studied coastline segments (Table 1; see Note 6 and Supplementary Table 4)."* (*note: 6.9% value calculated as *[n=20 20 km-long coastal segments where at least half of all well water level measurements lie below sea level] divided by [n=291 total 20 km-long coastal segments along the West Coast (see Table 1 second row, column entitled "Total number of segments (20-km-long) along coast" ] × 100%*

** identical to results presented in Supplementary Table 1 and results presented in the main text.

**Supplementary Table 5a.** Compilation of published coastal hydraulic gradient assessments. This Table 5a presents a compilation of studies documenting submarine groundwater discharges (see Supplementary Table 5b for a compilation of studies documenting a landward hydraulic gradient or seawater intrusion)

| Location title | Lat. | Lon. | Aquifer | Type | Aquifer depth range | Include in Supp. Fig. 19 ? | Result *** | Reference |
|---|---|---|---|---|---|---|---|---|
| Great South Bay, New York | 40.74 | -72.99 | None stated (shallow beach aquifer) | Shallow and likely unconfined (possible underlying confining layer) | <10 m | Yes | submarine groundwater discharge | Bokuniewicz, H., Pollock, M., Blum, J., & Wilson, R. (2004). Submarine ground water discharge and salt penetration across the sea floor. Groundwater, 42(7), 983-989. |
| Cape Henlopen, Delaware | 38.80 | -75.09 | None stated (shallow beach aquifer) | Shallow and likely unconfined (possible underlying confining layer) | Likely <10 m | Yes | submarine groundwater discharge | Hays, R. L., & Ullman, W. J. (2007). Direct determination of total and fresh groundwater discharge and nutrient loads from a sandy beachface at low tide (Cape Henlopen, Delaware). Limnology and oceanography, 52(1), 240-247. |
| Safety Harbor, Florida | 27.99 | -82.69 | None stated (shallow beach aquifer) | Shallow and likely unconfined | <28 m | Yes | submarine groundwater discharge | Kroeger, K. D., Swarzenski, P. W., Greenwood, W. J., & Reich, C. (2007). Submarine groundwater discharge to Tampa Bay: Nutrient fluxes and biogeochemistry of the coastal aquifer. Marine Chemistry, 104(1-2), 85-97. |
| Feather Sound, Florida | 27.91 | -82.66 | None stated (shallow beach aquifer) | Shallow and likely unconfined | <28 m | Yes | submarine groundwater discharge | Kroeger, K. D., Swarzenski, P. W., Greenwood, W. J., & Reich, C. (2007). Submarine groundwater discharge to Tampa Bay: Nutrient fluxes and biogeochemistry of the coastal aquifer. Marine Chemistry, 104(1-2), 85-97. |
| St. Petersburg, Florida | 27.77 | -82.63 | None stated (shallow beach aquifer) | Shallow and likely unconfined | <28 m | Yes | submarine groundwater discharge | Kroeger, K. D., Swarzenski, P. W., Greenwood, W. J., & Reich, C. (2007). Submarine groundwater discharge to Tampa Bay: Nutrient fluxes and biogeochemistry of the coastal aquifer. Marine Chemistry, 104(1-2), 85-97. |
| Pnellas Point, Florida | 27.70 | -82.64 | None stated (shallow beach aquifer) | Shallow and likely unconfined | <28 m | Yes | submarine groundwater discharge | Kroeger, K. D., Swarzenski, P. W., Greenwood, W. J., & Reich, C. (2007). Submarine groundwater discharge to Tampa Bay: Nutrient fluxes and biogeochemistry of the coastal aquifer. Marine Chemistry, 104(1-2), 85-97. |
| Waquoit Bay, Massachusetts | 41.58 | -70.52 | None stated (shallow aquifer) | Unconfined | — | Yes | submarine groundwater discharge | Mulligan, A. E., & Charette, M. A. (2006). Intercomparison of submarine groundwater discharge estimates from a sandy unconfined aquifer. Journal of Hydrology, 327(3-4), 411-425. |
| Holts Landing State Park, Delaware | 38.59 | -75.13 | None stated (shallow aquifer) | Likely unconfined | < 60 m | Yes | submarine groundwater discharge | Russoniello, C. J., Fernandez, C., Bratton, J. F., Banaszak, J. F., Krantz, D. E., Andres, A. S., Konikow, L. F., & Michael, H. A. (2013). Geologic effects on groundwater salinity and discharge into an estuary. Journal of hydrology, 498, 1-12. |

| Location title | Lat. | Lon. | Aquifer | Type | Aquifer depth range | Include in Supp. Fig. 19 ? | Result *** | Reference |
|---|---|---|---|---|---|---|---|---|
| Florida State Coastal and Marine Laboratory, Florida | 29.90 | -84.50 | Floridan aquifer system | Both unconfined and underyling confined | — | Yes | submarine groundwater discharge | Santos, I. R., Burnett, W. C., Chanton, J., Dimova, N., & Peterson, R. N. (2009). Land or ocean?: Assessing the driving forces of submarine groundwater discharge at a coastal site in the Gulf of Mexico. Journal of Geophysical Research: Oceans, 114(C4), C04012. |
| Huntington Beach, California | 33.64 | -117.97 | None stated (shallow beach aquifer) | Likely unconfined | — | Yes | submarine groundwater discharge | Boehm, A. B., Paytan, A., Shellenbarger, G. G., & Davis, K. A. (2006). Composition and flux of groundwater from a California beach aquifer: Implications for nutrient supply to the surf zone. Continental Shelf Research, 26(2), 269-282. |
| Santa Barbara, California | 34.41 | -119.69 | None stated (shallow beach aquifer) | Likely unconfined | — | Yes | submarine groundwater discharge | Swarzenski, P. W., & Izbicki, J. A. (2009). Coastal groundwater dynamics off Santa Barbara, California: Combining geochemical tracers, electromagnetic seepmeters, and electrical resistivity. Estuarine, Coastal and Shelf Science, 83(1), 77-89. |
| south of New Orleans, Louisiana | 29.31 | -89.97 | Coastal Lowland Aquifer system | Unconfined | — | Yes | submarine groundwater discharge | McCoy, C. A., Corbett, D. R., McKee, B. A., & Top, Z. (2007). An evaluation of submarine groundwater discharge along the continental shelf of Louisiana using a multiple tracer approach. Journal of Geophysical Research: Oceans, 112(C3). |
| Onslow Bay, North Carolina | 34.47 | -77.48 | Castle Hayne | Unconfined surficial aquifer and confined Castle Hayne | — | Yes | submarine groundwater discharge | McCoy, C. A., Corbett, D. R., Cable, J. E., & Spruill, R. K. (2007). Hydrogeological characterization of southeast coastal plain aquifers and groundwater discharge to Onslow Bay, North Carolina (USA). Journal of Hydrology, 339(3-4), 159-171. |
| Sunset State Beach, California | 36.88 | -121.83 | Aromas Sands and Purisima Aquifers | Unconfined | Likely <10 m | Yes | submarine groundwater discharge | Lecher, A. L., Mackey, K., Kudela, R., Ryan, J., Fisher, A., Murray, J., & Paytan, A. (2015). Nutrient loading through submarine groundwater discharge and phytoplankton growth in Monterey Bay, CA. Environmental science & technology, 49(11), 6665-6673. |
| Seabright Beach, California | 36.96 | -122.01 | — | — | — | Yes | submarine groundwater discharge | Lecher, A. L., Fisher, A. T., & Paytan, A. (2016). Submarine groundwater discharge in Northern Monterey Bay, California: Evaluation by mixing and mass balance models. Marine Chemistry, 179, 44-55. |
| Rio Del Mar Beach, California | 36.97 | -121.91 | — | — | — | Yes | submarine groundwater discharge | Lecher, A. L., Fisher, A. T., & Paytan, A. (2016). Submarine groundwater discharge in Northern Monterey Bay, California: Evaluation by mixing and mass balance models. Marine Chemistry, 179, 44-55. |
| Salinas River Beach, California | 36.79 | -121.79 | — | — | — | Yes | submarine groundwater discharge | Lecher, A. L., Fisher, A. T., & Paytan, A. (2016). Submarine groundwater discharge in Northern Monterey Bay, California: Evaluation by mixing and mass balance models. Marine Chemistry, 179, 44-55. |
| Stinson Beach, California | 37.90 | -122.65 | — | Unconfined | <32 m | Yes | submarine groundwater discharge | de Sieyes, N. R., Yamahara, K. M., Layton, B. A., Joyce, E. H., & Boehm, A. B. (2008). Submarine discharge of nutrient-enriched fresh groundwater at Stinson Beach, California is enhanced during neap tides. Limnology and Oceanography, 53(4), 1434-1445. |
| Little Lagoon, Alabama | 30.25 | -87.75 | — | Unconfined | — | Yes | submarine groundwater discharge | Su, N., Burnett, W. C., Eller, K. T., MacIntyre, H. L., Mortazavi, B., Liefer, J. D., & Novoveská, L. (2012). Radon and radium isotopes, |

| Location title | Lat. | Lon. | Aquifer | Type | Aquifer depth range | Include in Supp. Fig. 19 ? | Result *** | Reference |
|---|---|---|---|---|---|---|---|---|
| | | | | | | | | groundwater discharge and harmful algal blooms in Little Lagoon, Alabama. Interdisciplinary studies on environmental chemistry, 6, 329-337. |
| Little Lagoon, Alabama | 30.25 | -87.75 | — | Unconfined | — | Yes | submarine groundwater discharge | Su, N., Burnett, W. C., MacIntyre, H. L., Liefer, J. D., Peterson, R. N., & Viso, R. (2014). Natural radon and radium isotopes for assessing groundwater discharge into Little Lagoon, AL: implications for harmful algal blooms. Estuaries and coasts, 37(4), 893-910. |
| Turkey Point, Florida | 29.85 | -84.50 | — | — | — | Yes | submarine groundwater discharge | Moore, W. S. (2003). Sources and fluxes of submarine groundwater discharge delineated by radium isotopes. Biogeochemistry, 66(1-2), 75-93. |
| Turkey Point, Florida | 29.85 | -84.50 | — | Unconfined | <10m | Yes | submarine groundwater discharge | Smith, L., & Zawadzki, W. (2003). A hydrogeologic model of submarine groundwater discharge: Florida intercomparison experiment. Biogeochemistry, 66(1-2), 95-110. |
| Turkey Point, Florida | 29.85 | -84.50 | — | Unconfined | — | Yes | submarine groundwater discharge | Taniguchi, M., Burnett, W. C., Smith, C. F., Paulsen, R. J., O'rourke, D., Krupa, S. L., & Christoff, J. L. (2003). Spatial and temporal distributions of submarine groundwater discharge rates obtained from various types of seepage meters at a site in the Northeastern Gulf of Mexico. Biogeochemistry, 66(1-2), 35-53. |
| Hood Canal (study segment #1), Washington | 47.74 | -122.72 | — | — | — | Yes | submarine groundwater discharge | * Paulson, A. J., Konrad, C. P., Frans, L. M., Noble, M., Kendall, C., Josberger, E. G., Huffman, R. L., & Olsen, T. D. (2006). Freshwater and saline loads of dissolved inorganic nitrogen to Hood Canal and Lynch Cove, Western Washington. USGS Scientific Investigations Report 2006–5106 |
| Hood Canal (study segment #2), Washington | 47.64 | -122.81 | — | — | — | Yes | submarine groundwater discharge | * Paulson, A. J., Konrad, C. P., Frans, L. M., Noble, M., Kendall, C., Josberger, E. G., Huffman, R. L., & Olsen, T. D. (2006). Freshwater and saline loads of dissolved inorganic nitrogen to Hood Canal and Lynch Cove, Western Washington. USGS Scientific Investigations Report 2006–5106 |
| Hood Canal (study segment #3), Washington | 47.59 | -122.92 | — | — | — | Yes | submarine groundwater discharge | * Paulson, A. J., Konrad, C. P., Frans, L. M., Noble, M., Kendall, C., Josberger, E. G., Huffman, R. L., & Olsen, T. D. (2006). Freshwater and saline loads of dissolved inorganic nitrogen to Hood Canal and Lynch Cove, Western Washington. USGS Scientific Investigations Report 2006–5106 |
| Hood Canal (study segment #4), Washington | 47.52 | -123.02 | — | — | — | Yes | submarine groundwater discharge | * Paulson, A. J., Konrad, C. P., Frans, L. M., Noble, M., Kendall, C., Josberger, E. G., Huffman, R. L., & Olsen, T. D. (2006). Freshwater and saline loads of dissolved inorganic nitrogen to Hood Canal and Lynch Cove, Western Washington. USGS Scientific Investigations Report 2006–5106 |
| Hood Canal (study segment #5), Washington | 47.44 | -123.08 | — | — | — | Yes | submarine groundwater discharge | * Paulson, A. J., Konrad, C. P., Frans, L. M., Noble, M., Kendall, C., Josberger, E. G., Huffman, R. L., & Olsen, T. D. (2006). Freshwater and saline loads of dissolved inorganic nitrogen to Hood Canal and Lynch |

| Location title | Lat. | Lon. | Aquifer | Type | Aquifer depth range | Include in Supp. Fig. 19 ? | Result *** | Reference |
|---|---|---|---|---|---|---|---|---|
| | | | | | | | | Cove, Western Washington. USGS Scientific Investigations Report 2006–5106 |
| Hood Canal (study segment #6), Washington | 47.37 | -123.07 | — | — | — | Yes | submarine groundwater discharge | * Paulson, A. J., Konrad, C. P., Frans, L. M., Noble, M., Kendall, C., Josberger, E. G., Huffman, R. L., & Olsen, T. D. (2006). Freshwater and saline loads of dissolved inorganic nitrogen to Hood Canal and Lynch Cove, Western Washington. USGS Scientific Investigations Report 2006–5106 |
| Hood Canal (study segment #7), Washington | 47.41 | -122.94 | — | — | — | Yes | submarine groundwater discharge | * Paulson, A. J., Konrad, C. P., Frans, L. M., Noble, M., Kendall, C., Josberger, E. G., Huffman, R. L., & Olsen, T. D. (2006). Freshwater and saline loads of dissolved inorganic nitrogen to Hood Canal and Lynch Cove, Western Washington. USGS Scientific Investigations Report 2006–5106 |
| Hood Canal (study segment #8), Washington | 47.44 | -122.87 | — | — | — | Yes | submarine groundwater discharge | * Paulson, A. J., Konrad, C. P., Frans, L. M., Noble, M., Kendall, C., Josberger, E. G., Huffman, R. L., & Olsen, T. D. (2006). Freshwater and saline loads of dissolved inorganic nitrogen to Hood Canal and Lynch Cove, Western Washington. USGS Scientific Investigations Report 2006–5106 |
| Hood Canal (study segment #9), Washington | 47.38 | -122.96 | — | — | — | Yes | submarine groundwater discharge | * Paulson, A. J., Konrad, C. P., Frans, L. M., Noble, M., Kendall, C., Josberger, E. G., Huffman, R. L., & Olsen, T. D. (2006). Freshwater and saline loads of dissolved inorganic nitrogen to Hood Canal and Lynch Cove, Western Washington. USGS Scientific Investigations Report 2006–5106 |
| Hood Canal (study segment #10), Washington | 47.36 | -123.16 | — | — | — | Yes | submarine groundwater discharge | * Paulson, A. J., Konrad, C. P., Frans, L. M., Noble, M., Kendall, C., Josberger, E. G., Huffman, R. L., & Olsen, T. D. (2006). Freshwater and saline loads of dissolved inorganic nitrogen to Hood Canal and Lynch Cove, Western Washington. USGS Scientific Investigations Report 2006–5106 |
| Hood Canal (study segment #11), Washington | 47.45 | -123.12 | — | — | — | Yes | submarine groundwater discharge | * Paulson, A. J., Konrad, C. P., Frans, L. M., Noble, M., Kendall, C., Josberger, E. G., Huffman, R. L., & Olsen, T. D. (2006). Freshwater and saline loads of dissolved inorganic nitrogen to Hood Canal and Lynch Cove, Western Washington. USGS Scientific Investigations Report 2006–5106 |
| Hood Canal (study segment #12), Washington | 47.47 | -123.10 | — | — | — | Yes | submarine groundwater discharge | * Paulson, A. J., Konrad, C. P., Frans, L. M., Noble, M., Kendall, C., Josberger, E. G., Huffman, R. L., & Olsen, T. D. (2006). Freshwater and saline loads of dissolved inorganic nitrogen to Hood Canal and Lynch Cove, Western Washington. USGS Scientific Investigations Report 2006–5106 |
| Hood Canal (study segment #13), Washington | 47.51 | -123.06 | — | — | — | Yes | submarine groundwater discharge | * Paulson, A. J., Konrad, C. P., Frans, L. M., Noble, M., Kendall, C., Josberger, E. G., Huffman, R. L., & Olsen, T. D. (2006). Freshwater and saline loads of dissolved inorganic nitrogen to Hood Canal and Lynch |

| Location title | Lat. | Lon. | Aquifer | Type | Aquifer depth range | Include in Supp. Fig. 19 ? | Result *** | Reference |
|---|---|---|---|---|---|---|---|---|
| | | | | | | | | Cove, Western Washington. USGS Scientific Investigations Report 2006–5106 |
| Hood Canal (study segment #14), Washington | 47.54 | -123.04 | — | — | — | Yes | submarine groundwater discharge | * Paulson, A. J., Konrad, C. P., Frans, L. M., Noble, M., Kendall, C., Josberger, E. G., Huffman, R. L., & Olsen, T. D. (2006). Freshwater and saline loads of dissolved inorganic nitrogen to Hood Canal and Lynch Cove, Western Washington. USGS Scientific Investigations Report 2006–5106 |
| Hood Canal (study segment #15), Washington | 47.56 | -123.04 | — | — | — | Yes | submarine groundwater discharge | * Paulson, A. J., Konrad, C. P., Frans, L. M., Noble, M., Kendall, C., Josberger, E. G., Huffman, R. L., & Olsen, T. D. (2006). Freshwater and saline loads of dissolved inorganic nitrogen to Hood Canal and Lynch Cove, Western Washington. USGS Scientific Investigations Report 2006–5106 |
| Hood Canal (study segment #16), Washington | 47.59 | -123.01 | — | — | — | Yes | submarine groundwater discharge | * Paulson, A. J., Konrad, C. P., Frans, L. M., Noble, M., Kendall, C., Josberger, E. G., Huffman, R. L., & Olsen, T. D. (2006). Freshwater and saline loads of dissolved inorganic nitrogen to Hood Canal and Lynch Cove, Western Washington. USGS Scientific Investigations Report 2006–5106 |
| Hood Canal (study segment #17), Washington | 47.63 | -122.96 | — | — | — | Yes | submarine groundwater discharge | * Paulson, A. J., Konrad, C. P., Frans, L. M., Noble, M., Kendall, C., Josberger, E. G., Huffman, R. L., & Olsen, T. D. (2006). Freshwater and saline loads of dissolved inorganic nitrogen to Hood Canal and Lynch Cove, Western Washington. USGS Scientific Investigations Report 2006–5106 |
| Hood Canal (study segment #18), Washington | 47.67 | -122.91 | — | — | — | Yes | submarine groundwater discharge | * Paulson, A. J., Konrad, C. P., Frans, L. M., Noble, M., Kendall, C., Josberger, E. G., Huffman, R. L., & Olsen, T. D. (2006). Freshwater and saline loads of dissolved inorganic nitrogen to Hood Canal and Lynch Cove, Western Washington. USGS Scientific Investigations Report 2006–5106 |
| Hood Canal (study segment #19), Washington | 47.72 | -122.90 | — | — | — | Yes | submarine groundwater discharge | * Paulson, A. J., Konrad, C. P., Frans, L. M., Noble, M., Kendall, C., Josberger, E. G., Huffman, R. L., & Olsen, T. D. (2006). Freshwater and saline loads of dissolved inorganic nitrogen to Hood Canal and Lynch Cove, Western Washington. USGS Scientific Investigations Report 2006–5106 |
| Hood Canal (study segment #20), Washington | 47.78 | -122.87 | — | — | — | Yes | submarine groundwater discharge | * Paulson, A. J., Konrad, C. P., Frans, L. M., Noble, M., Kendall, C., Josberger, E. G., Huffman, R. L., & Olsen, T. D. (2006). Freshwater and saline loads of dissolved inorganic nitrogen to Hood Canal and Lynch Cove, Western Washington. USGS Scientific Investigations Report 2006–5106 |
| Hood Canal (study segment #21), Washington | 47.80 | -122.83 | — | — | — | Yes | submarine groundwater discharge | * Paulson, A. J., Konrad, C. P., Frans, L. M., Noble, M., Kendall, C., Josberger, E. G., Huffman, R. L., & Olsen, T. D. (2006). Freshwater and saline loads of dissolved inorganic nitrogen to Hood Canal and Lynch |

| Location title | Lat. | Lon. | Aquifer | Type | Aquifer depth range | Include in Supp. Fig. 19 ? | Result *** | Reference |
|---|---|---|---|---|---|---|---|---|
| | | | | | | | | Cove, Western Washington. USGS Scientific Investigations Report 2006–5106 |
| Hood Canal (study segment #22), Washington | 47.75 | -122.79 | — | — | — | Yes | submarine groundwater discharge | * Paulson, A. J., Konrad, C. P., Frans, L. M., Noble, M., Kendall, C., Josberger, E. G., Huffman, R. L., & Olsen, T. D. (2006). Freshwater and saline loads of dissolved inorganic nitrogen to Hood Canal and Lynch Cove, Western Washington. USGS Scientific Investigations Report 2006–5106 |
| Hood Canal (study segment #23), Washington | 47.87 | -122.70 | — | — | — | Yes | submarine groundwater discharge | * Paulson, A. J., Konrad, C. P., Frans, L. M., Noble, M., Kendall, C., Josberger, E. G., Huffman, R. L., & Olsen, T. D. (2006). Freshwater and saline loads of dissolved inorganic nitrogen to Hood Canal and Lynch Cove, Western Washington. USGS Scientific Investigations Report 2006–5106 |
| Little Quilcene, Washington | 47.81 | -122.86 | — | — | — | Yes | submarine groundwater discharge | * Paulson, A. J., Konrad, C. P., Frans, L. M., Noble, M., Kendall, C., Josberger, E. G., Huffman, R. L., & Olsen, T. D. (2006). Freshwater and saline loads of dissolved inorganic nitrogen to Hood Canal and Lynch Cove, Western Washington. USGS Scientific Investigations Report 2006–5106 |
| Big Quilcene, Washington | 47.80 | -122.87 | — | — | — | Yes | submarine groundwater discharge | * Paulson, A. J., Konrad, C. P., Frans, L. M., Noble, M., Kendall, C., Josberger, E. G., Huffman, R. L., & Olsen, T. D. (2006). Freshwater and saline loads of dissolved inorganic nitrogen to Hood Canal and Lynch Cove, Western Washington. USGS Scientific Investigations Report 2006–5106 |
| Spencer, Washington | 47.75 | -122.87 | — | — | — | Yes | submarine groundwater discharge | * Paulson, A. J., Konrad, C. P., Frans, L. M., Noble, M., Kendall, C., Josberger, E. G., Huffman, R. L., & Olsen, T. D. (2006). Freshwater and saline loads of dissolved inorganic nitrogen to Hood Canal and Lynch Cove, Western Washington. USGS Scientific Investigations Report 2006–5106 |
| Jackson, Washington | 47.74 | -122.88 | — | — | — | Yes | submarine groundwater discharge | * Paulson, A. J., Konrad, C. P., Frans, L. M., Noble, M., Kendall, C., Josberger, E. G., Huffman, R. L., & Olsen, T. D. (2006). Freshwater and saline loads of dissolved inorganic nitrogen to Hood Canal and Lynch Cove, Western Washington. USGS Scientific Investigations Report 2006–5106 |
| Union, Washington | 47.44 | -122.84 | — | — | — | Yes | submarine groundwater discharge | * Paulson, A. J., Konrad, C. P., Frans, L. M., Noble, M., Kendall, C., Josberger, E. G., Huffman, R. L., & Olsen, T. D. (2006). Freshwater and saline loads of dissolved inorganic nitrogen to Hood Canal and Lynch Cove, Western Washington. USGS Scientific Investigations Report 2006–5106 |
| Mission, Washington | 47.45 | -122.84 | — | — | — | Yes | submarine groundwater discharge | * Paulson, A. J., Konrad, C. P., Frans, L. M., Noble, M., Kendall, C., Josberger, E. G., Huffman, R. L., & Olsen, T. D. (2006). Freshwater and saline loads of dissolved inorganic nitrogen to Hood Canal and Lynch |

| Location title | Lat. | Lon. | Aquifer | Type | Aquifer depth range | Include in Supp. Fig. 19 ? | Result *** | Reference |
|---|---|---|---|---|---|---|---|---|
| | | | | | | | | Cove, Western Washington. USGS Scientific Investigations Report 2006–5106 |
| Tahuya, Washington | 47.38 | -123.04 | — | — | — | Yes | submarine groundwater discharge | * Paulson, A. J., Konrad, C. P., Frans, L. M., Noble, M., Kendall, C., Josberger, E. G., Huffman, R. L., & Olsen, T. D. (2006). Freshwater and saline loads of dissolved inorganic nitrogen to Hood Canal and Lynch Cove, Western Washington. USGS Scientific Investigations Report 2006–5106 |
| Eagle, Washington | 47.47 | -123.09 | — | — | — | Yes | submarine groundwater discharge | * Paulson, A. J., Konrad, C. P., Frans, L. M., Noble, M., Kendall, C., Josberger, E. G., Huffman, R. L., & Olsen, T. D. (2006). Freshwater and saline loads of dissolved inorganic nitrogen to Hood Canal and Lynch Cove, Western Washington. USGS Scientific Investigations Report 2006–5106 |
| Finch, Washington | 47.40 | -123.15 | — | — | — | Yes | submarine groundwater discharge | * Paulson, A. J., Konrad, C. P., Frans, L. M., Noble, M., Kendall, C., Josberger, E. G., Huffman, R. L., & Olsen, T. D. (2006). Freshwater and saline loads of dissolved inorganic nitrogen to Hood Canal and Lynch Cove, Western Washington. USGS Scientific Investigations Report 2006–5106 |
| Fulton, Washington | 47.49 | -123.08 | — | — | — | Yes | submarine groundwater discharge | * Paulson, A. J., Konrad, C. P., Frans, L. M., Noble, M., Kendall, C., Josberger, E. G., Huffman, R. L., & Olsen, T. D. (2006). Freshwater and saline loads of dissolved inorganic nitrogen to Hood Canal and Lynch Cove, Western Washington. USGS Scientific Investigations Report 2006–5106 |
| Jorsted, Washington | 47.52 | -123.05 | — | — | — | Yes | submarine groundwater discharge | * Paulson, A. J., Konrad, C. P., Frans, L. M., Noble, M., Kendall, C., Josberger, E. G., Huffman, R. L., & Olsen, T. D. (2006). Freshwater and saline loads of dissolved inorganic nitrogen to Hood Canal and Lynch Cove, Western Washington. USGS Scientific Investigations Report 2006–5106 |
| Lilliwaup, Washington | 47.47 | -123.12 | — | — | — | Yes | submarine groundwater discharge | * Paulson, A. J., Konrad, C. P., Frans, L. M., Noble, M., Kendall, C., Josberger, E. G., Huffman, R. L., & Olsen, T. D. (2006). Freshwater and saline loads of dissolved inorganic nitrogen to Hood Canal and Lynch Cove, Western Washington. USGS Scientific Investigations Report 2006–5106 |
| Waketickeh, Washington | 47.56 | -123.03 | — | — | — | Yes | submarine groundwater discharge | * Paulson, A. J., Konrad, C. P., Frans, L. M., Noble, M., Kendall, C., Josberger, E. G., Huffman, R. L., & Olsen, T. D. (2006). Freshwater and saline loads of dissolved inorganic nitrogen to Hood Canal and Lynch Cove, Western Washington. USGS Scientific Investigations Report 2006–5106 |
| Long Island, New York | 40.97 | -73.09 | — | — | — | Yes | submarine groundwater discharge | Tamborski, J. J., Rogers, A. D., Bokuniewicz, H. J., Cochran, J. K., & Young, C. R. (2015). Identification and quantification of diffuse fresh submarine groundwater discharge via airborne thermal infrared remote sensing. Remote Sensing of Environment, 171, 202-217. |

**Supplementary Table 5b.** Compilation of published coastal hydraulic gradient assessments. This Table 5b presents a compilation of studies documenting a landward hydraulic gradient or seawater intrusion (see Supplementary Table 5a for a compilation of studies documenting submarine groundwater discharges)

| Location title | Lat. | Lon. | Aquifer | Type | Aquifer depth range | Include in Supp. Fig. 19 ? | Result *** | Reference |
|---|---|---|---|---|---|---|---|---|
| Long Beach, California | 33.78 | -118.19 | Sunnyside Aquifer, Silverado Aquifer, Lynwood aquifer | Unconfined-to-confined | ~800m | Yes | Landward gradient | Nishikawa, T., Siade, A. J., Reichard, E. G., Ponti, D. J., Canales, A. G., & Johnson, T. A. (2009). Stratigraphic controls on seawater intrusion and implications for groundwater management, Dominguez Gap area of Los Angeles, California, USA. Hydrogeology journal, 17(7), 1699. |
| Hilton Head, South Carolina | 32.22 | -80.75 | Upper Floridan Aquifer | — | — | Yes | Seawater intrusion | Payne, D. F. (2010). Effects of climate change on saltwater intrusion at Hilton Head Island , S C. U.S.A. (Proceedings of SWIM21 - 21st Salt Water Intrusion Meeting: June 21-26, 2010). Available at: http://www.swim-site.nl/pdf/swim21/pages_293_296.pdf |
| Parajo Valley, California | 36.89 | -121.83 | Aromas Sand | Semi-confined/Confined | <~150 m | Yes | Seawater intrusion | Hanson, R. T. (2003). Geohydrologic framework of recharge and seawater intrusion in the Pajaro Valley, Santa Cruz and Monterey Counties, California. Water Resources Investigations Report 03-4096. |
| Monterey Bay, California | 36.73 | -121.75 | "180-Foot Aquifer", "400-Foot Aquifer", "900-foot Aquifer" | Semi-confined to Confined | <~300 m | Yes | Seawater intrusion | Monterey CountyWater Resource Agency (2006). Monterey County Groundwater Management Plan. 78 pp. Accessed February 11, 2020 via: https://water.ca.gov/LegacyFiles/groundwater/docs/GWMP/CC-3_MontereyCoWRA_GWMP_2006.pdf |
| Monterey Bay, California | 36.65 | -121.83 | "180-Foot Aquifer" | Semi-confined to Confined | <~100 m | Yes | Seawater intrusion | Goebel, M., Pidlisecky, A., & Knight, R. (2017). Resistivity imaging reveals complex pattern of saltwater intrusion along Monterey coast. Journal of hydrology, 551, 746-755. |
| Oxnard Plain, California | 34.20 | -119.23 | Upper Aquifer System and Lower Aquifer System | — | — | Yes | Seawater intrusion | Martin, J. N. (2013) Central Coast Groundwater: Seawater Intrusion and Other Issues. Report Commissioned by the California Water Foundation. 27 pp. Accessed February 11, 2020 via: https://water.ca.gov/LegacyFiles/waterplan/docs/cwpu2013/Final/vol4/groundwater/11Central_Coast_Groundwater_Seawater_Intrusion.pdf |
| New Orleans, Louisiana | 30.00 | -90.24 | Gonzales-New Orleans Aquifer | — | <250m | Yes | Seawater intrusion | Prakken, L. B. (2009) Groundwater Resources in the New Oreleans Area 2008. Louisiana Department of Transportation and Development Report. Technical Report No. 80. See also: https://pubs.usgs.gov/wri/1995/4169/plate-1.pdf |
| Baldwin County, Alabama | 30.33 | -87.65 | Beach Sand Aquifer ("A1"), Gulf Shores Aquifer ("A2") and Deep Miocene Aquifer ("A3") | Unconfined A1; Unconfined-to-Semi-Confined A2 | <300 m | Yes | Seawater intrusion | Murgulet, D., & Tick, G. (2008). The extent of saltwater intrusion in southern Baldwin County, Alabama. Environmental Geology, 55(6), 1235-1245. |

| Location title | Lat. | Lon. | Aquifer | Type | Aquifer depth range | Include in Supp. Fig. 19 ? | Result *** | Reference |
|---|---|---|---|---|---|---|---|---|
| Miami, Florida | 25.76 | -80.20 | Biscayne Aquifer | Unconfined | <50 m | Yes | Seawater intrusion | Prinos, S. T., Wacker, M. A., Cunningham, K. J., & Fitterman, D. V. (2014). Origins and delineation of saltwater intrusion in the Biscayne aquifer and changes in the distribution of saltwater in Miami-Dade County, Florida. US Geological Survey Report No. 2014-5025. https://pubs.er.usgs.gov/publication/sir20145025 |
| Tequesta Well Field, Florida | 26.96 | -80.11 | — | — | <75 m | Yes | Seawater intrusion | Scott, W.B., Land, L.F., and Rodis, H.G., 1977, Saltwater intrusion in the shallow aquifer in Palm Beach and Martin Counties, Florida: U.S. Geological Survey Water-Resources Investigations Report 76-135, 1 sheet. |
| Boca Raton, Florida | 26.36 | -80.11 | — | — | — | Yes | Seawater intrusion | Renken R. A., Dixon J., Koehmstedt, J., Ishman, S., Lietz, A. C., Marella, R.L., Telis, P., Rogers, J., Memberg, S. (2005). Impact of anthropogenic development on coastal ground-water hydrology in southeastern Florida, 1900–2000. US Geol Surv Circ 1275 |
| Sunny Isles Well Field, Florida | 25.93 | -80.17 | — | — | — | Yes | Seawater intrusion | Renken R. A., Dixon J., Koehmstedt, J., Ishman, S., Lietz, A. C., Marella, R.L., Telis, P., Rogers, J., Memberg, S. (2005). Impact of anthropogenic development on coastal ground-water hydrology in southeastern Florida, 1900–2000. US Geol Surv Circ 1275 |
| East Side Well Field, Florida | 25.90 | -80.19 | — | — | ~100m | Yes | Seawater intrusion | Merritt, M. (1996). Assessment of Saltwater intrusion in Southern Coastal Broward County. U.S. Geological Survey Water Resources Investirgations Report 96-4221. Accessed February 11, 2020 via https://www.nrc.gov/docs/ML1428/ML14287A522.pdf |
| Hallandale Well Field, Florida | 25.99 | -80.16 | — | — | <250m | Yes | Seawater intrusion | Renken R. A., Dixon J., Koehmstedt, J., Ishman, S., Lietz, A. C., Marella, R.L., Telis, P., Rogers, J., Memberg, S. (2005). Impact of anthropogenic development on coastal ground-water hydrology in southeastern Florida, 1900–2000. US Geol Surv Circ 1275 |
| Savannah, Georgia | 32.07 | -81.07 | Upper Floridan Aquifer | Confined | ~20-70m | Yes | Landward gradient | Foyle, A. M., Henry, V. J., & Alexander, C. R. (2002). Mapping the threat of seawater intrusion in a regional coastal aquifer–aquitard system in the southeastern United States. Environmental Geology, 43(1-2), 151-159. |
| Virginia Beach, Virginia | 36.87 | -76.09 | Columbia Aquifer, Yorktown-Eastover Aquifer | Generally unconfined (Columbia Aquifer); Generally confined (Yorktown-Eastover) | <~60m | Yes | Seawater intrusion | Smith, B. S., & Harlow Jr, G. E. (2002). Conceptual hydrogeologic framework of the shallow aquifer system at Virginia Beach, Virginia. Water-Resources Investigations Report, 1, 4262. |
| southern Louisiana | 29.99 | -92.34 | Chicot Aquifer | Unconfined in north, confined in south | <~220 m | Yes | Seawater intrusion | Borrok, D. M., & Broussard III, W. P. (2016). Long-term geochemical evaluation of the coastal Chicot aquifer system, Louisiana, USA. Journal of Hydrology, 533, 320-331. |

| Location title | Lat. | Lon. | Aquifer | Type | Aquifer depth range | Include in Supp. Fig. 19 ? | Result *** | Reference |
|---|---|---|---|---|---|---|---|---|
| Myrtle Beach, South Carolina | 33.70 | -78.89 | Surficial Aquifer; Floridan Aquifer System; Tertiary Sand Aquifer; Black Creek Aquifer, Middendorf Aquifer; Cape Fear Aquifer. | Unconfined (e.g., Surficial Aquifer) to confined (deeper aquifers) | <1,000 m | Yes | Landward gradient | Aucott, W.R. (1996). Hydrology of the Southeastern Coastal Plain aquifer system in South Carolina and parts of Georgia and North Carolina: U.S. Geological Survey Professional Paper 1410-E, 83 p. https://pubs.usgs.gov/pp/1410e/report.pdf |
| Savannah, Georgia | 32.07 | -81.07 | Floridan Aquifer | — | — | Yes | Landward gradient | Bush, P.W., and Johnston, R.H., 1988, Ground-water hydraulics, regional flow, and ground-water development of the Floridan aquifer system in Florida and in parts of Georgia, South Carolina, and Alabama: U.S. Geological Survey Professional Paper 1403-C, 80 p. https://pubs.usgs.gov/pp/1403c/report.pdf |
| Daytona Beach, Florida | 29.18 | -81.04 | Floridan Aquifer | — | — | Yes | Seawater intrusion | Bush, P.W., and Johnston, R.H., 1988, Ground-water hydraulics, regional flow, and ground-water development of the Floridan aquifer system in Florida and in parts of Georgia, South Carolina, and Alabama: U.S. Geological Survey Professional Paper 1403-C, 80 p. https://pubs.usgs.gov/pp/1403c/report.pdf |
| Brunswick, Georgia | 31.15 | -81.49 | Floridan Aquifer | — | — | No | Saltwater intrusion ** | Bush, P.W., and Johnston, R.H., 1988, Ground-water hydraulics, regional flow, and ground-water development of the Floridan aquifer system in Florida and in parts of Georgia, South Carolina, and Alabama: U.S. Geological Survey Professional Paper 1403-C, 80 p. https://pubs.usgs.gov/pp/1403c/report.pdf |
| Lewes, Delaware | 38.78 | -75.15 | Quaternary Aquifer | — | — | Yes | Seawater intrusion | Cushing, E.M., Kantrowitz, I.H., and Taylor, K.R., 1973, Water resources of the Delmarva Peninsula: U.S. Geological Survey Professional Paper 822, 58 p. https://pubs.usgs.gov/pp/0822/report.pdf |
| Cambridge, Delaware | 38.57 | -76.08 | Piney Point Aquifer | — | <500m | Yes | Landward gradient | Cushing, E.M., Kantrowitz, I.H., and Taylor, K.R., 1973, Water resources of the Delmarva Peninsula: U.S. Geological Survey Professional Paper 822, 58 p. https://pubs.usgs.gov/pp/0822/report.pdf |
| Dover, Delaware | 39.16 | -75.51 | Piney Point Aquifer | — | <500m | Yes | Landward gradient | Cushing, E.M., Kantrowitz, I.H., and Taylor, K.R., 1973, Water resources of the Delmarva Peninsula: U.S. Geological Survey Professional Paper 822, 58 p. https://pubs.usgs.gov/pp/0822/report.pdf (see also https://agu.confex.com/agu/fm19/meetingapp.cgi/Paper/617689) |
| Cape May, New Jersey | 38.94 | -74.91 | Cohansey Sand (equivalent to Upper | — | — | Yes | Seawater intrusion | Knobel, L.L., Chapelle, F.H., & Meisler, H. Geochemistry of the northern Atlantic Coastal Plain aquifer system: U.S. Geological Survey Professional Paper 1404-L, 57 pp. (1998). Available via: https://pubs.usgs.gov/pp/1404l/report.pdf |

| Location title | Lat. | Lon. | Aquifer | Type | Aquifer depth range | Include in Supp. Fig. 19 ? | Result *** | Reference |
|---|---|---|---|---|---|---|---|---|
| | | | Chesapeake Aquifer) | | | | | |
| Baltimore, Maryland | 39.29 | -76.61 | Patuxent Aquifer | Confined | ~30-70m | Yes | Seawater intrusion | Chapelle, F.H., 1985, Hydrogeology, digital solute-transport simulation, and geochemistry of the Lower Cretaceous aquifer system near Baltimore, Maryland, with a section on Well records, pumpage information and other supplemental data by T.M. Kean: Maryland Geological Survey Report of Investigations 43, 120 p. https://pubs.er.usgs.gov/publication/70114204 |
| Bethany Beach, Delaware | 38.54 | -75.06 | Columbia Aquifer (Pleistocene Aquifer) | Unconfined | 20-55m | Yes | Seawater intrusion | Phelan, D. J. (1987) Water levels, chloride concentrations, and pumpage in the coastal aquifers of Delaware and Maryland. Water-Resources Investigations Report 87-4229, 106 pp. |
| Fernandina Beach, Florida | 30.66 | -81.45 | Floridan Aquifer | Confined | — | Yes | Landward gradient | Fairchild, R.W., & Bentley, C.B. (1977). Saline-water intrusion in the Floridan aquifer in the Fernandina Beach area, Nassau County, Florida: U.S. Geological Survey Water-Resources Investigations Report 77-32, 27 p. https://pubs.er.usgs.gov/publication/wri7732 |
| Salem, New Jersey | 39.57 | -75.47 | Potomac-Raritan-Magothy aquifer system (PRM) | Unconfined | <~300 m | Yes | Landward gradient | Cauller, S.J., Carleton, G.B., and Storck, M.J., 1999, Hydrogeology of, water withdrawal from, and water levels and chloride concentrations in the major Coastal Plain aquifers of Gloucester and Salem Counties, New Jersey: U.S. Geological Survey Water-Resources Investigations Report 98-4136, 123 p. https://pubs.usgs.gov/wri/wri98-4136/ |
| Paulsboro and Gibbstown, New Jersey | 39.83 | -75.26 | Potomac-Raritan-Magothy aquifer system (PRM) | Unconfined | <~300 m | Yes | Seawater intrusion | Ervin, E.M, Voronin, L.M., and Fusillo, T.V., 1994, Water quality of the Potomac-Raritan-Magothy aquifer system in the coastal plain, west-central New Jersey: U.S. Geological Survey Water-Resources Investigations Report 94-4113, 114 p |
| Cape May, New Jersey | 39.05 | -74.85 | Holly Beach sand | Unconfined | <25 m | Yes | Seawater intrusion | Lacombe, P. J., & Carleton, G. B. (2002). Hydrogeologic framework, availability of water supplies, and saltwater intrusion, Cape May County, New Jersey (No. 1). Water-Resources Investigations Report 01-4246 US Department of the Interior, US Geological Survey.https://pubs.usgs.gov/wri/wri014246/ |
| Cape May, New Jersey | 39.05 | -74.85 | Esturine sand | Confined | 40-70 m | Yes | Seawater intrusion | Lacombe, P. J., & Carleton, G. B. (2002). Hydrogeologic framework, availability of water supplies, and saltwater intrusion, Cape May County, New Jersey (No. 1). Water-Resources Investigations Report 01-4246 US Department of the Interior, US Geological Survey.https://pubs.usgs.gov/wri/wri014246/ |
| Cape May, New Jersey | 39.05 | -74.85 | Cohancy sand | Confined | 25-130 m | Yes | Seawater intrusion | Lacombe, P. J., & Carleton, G. B. (2002). Hydrogeologic framework, availability of water supplies, and saltwater intrusion, Cape May County, New Jersey (No. 1). Water-Resources Investigations Report 01-4246 US Department of the Interior, US Geological Survey.https://pubs.usgs.gov/wri/wri014246/ |

| Location title | Lat. | Lon. | Aquifer | Type | Aquifer depth range | Include in Supp. Fig. 19 ? | Result *** | Reference |
|---|---|---|---|---|---|---|---|---|
| Cape May, New Jersey | 39.05 | -74.85 | Rio Grande | Confined | 50-190 m | Yes | Seawater intrusion | Lacombe, P. J., & Carleton, G. B. (2002). Hydrogeologic framework, availability of water supplies, and saltwater intrusion, Cape May County, New Jersey (No. 1). Water-Resources Investigations Report 01-4246 US Department of the Interior, US Geological Survey.https://pubs.usgs.gov/wri/wri014246/ |
| Cape May, New Jersey | 39.05 | -74.85 | AC 800 foot sand | Confined | 200-320 m | Yes | Seawater intrusion | Lacombe, P. J., & Carleton, G. B. (2002). Hydrogeologic framework, availability of water supplies, and saltwater intrusion, Cape May County, New Jersey (No. 1). Water-Resources Investigations Report 01-4246 US Department of the Interior, US Geological Survey.https://pubs.usgs.gov/wri/wri014246/ |
| Atlantic City, New Jersey | 39.40 | -74.51 | Atlantic City 800 FT Sand | Confined | 200-250 m | Yes | Seawater intrusion | McAuley, S. D., Barringer, J. L., Paulachok, G. N., Clark, J. S., & Zapecza, O. S. (2001). Groundwater flow and quality in the Atlantic City 800 Foot Sand, New Jersey. New Jersey Geological Survey Report 41. https://pubs.er.usgs.gov/publication/70114185 |
| Camden, New Jersey | 39.96 | -75.09 | Potomac-Raritan-Magothy aquifer system (PRM) | Unconfined | <260 m | Yes | Seawater intrusion | Navoy, A. S., Voronin, L. M., & Modica, E. (2005). Vulnerability of production wells in the Potomac-Raritan-Magothy aquifer system to saltwater intrusion from the Delaware River in Camden, Gloucester, and Salem Counties, New Jersey. US Department of the Interior, US Geological Survey Scientific Investigations Report 2004-5096 https://pubs.er.usgs.gov/publication/sir20045096 |
| Northern New Jersey Coastal Plain, New Jersey | 40.33 | -74.13 | Magothy formation(upper aquifer) | Confined | 240-340 m | Yes | Seawater intrusion | Pucci Jr, A. A., Pope, D. A., & Gronberg, J. M. Hydrogeology, simulation of regional ground-water flow, and saltwater intrusion, Potomac-Raritan-Magothy aquifer system, northern coastal plain of New Jersey. New Jersey Geological Survey Report GSR36 (1994). Available via: https://pubs.er.usgs.gov/publication/70159214 |
| Northern New Jersey Coastal Plain, New Jersey | 40.33 | -74.13 | Raritan formation (middle aquifer) | Confined | 360-400 m | Yes | Seawater intrusion | Pucci Jr, A. A., Pope, D. A., & Gronberg, J. M. Hydrogeology, simulation of regional ground-water flow, and saltwater intrusion, Potomac-Raritan-Magothy aquifer system, northern coastal plain of New Jersey. New Jersey Geological Survey Report GSR36 (1994). Available via: https://pubs.er.usgs.gov/publication/70159214 |
| Southern Hills regional aquifer system, Baton Rouge Area, Louisiana | 30.35 | -91.13 | 1500-foot Sand | Confined | ~460m | Yes | Seawater intrusion | Heywood, C.E., Lindaman, M., and Lovelace, J.K., 2019, Simulation of groundwater flow and chloride transport in the "1,500-foot" sand, "2,400-foot" sand, and "2,800-foot" sand of the Baton Rouge area, Louisiana: U.S. Geological Survey Scientific Investigations Report 2019–5102, 49 p., https://doi.org/10.3133/sir20195102. |
| Southern Hills regional aquifer system, Baton Rouge Area, Louisiana | 30.35 | -91.13 | 2400-foot-Sand | Confined | ~730 m | Yes | Seawater intrusion | Heywood, C.E., Lindaman, M., and Lovelace, J.K., 2019, Simulation of groundwater flow and chloride transport in the "1,500-foot" sand, "2,400-foot" sand, and "2,800-foot" sand of the Baton Rouge area, Louisiana: U.S. Geological Survey Scientific Investigations Report 2019–5102, 49 p., https://doi.org/10.3133/sir20195102. |

| Location title | Lat. | Lon. | Aquifer | Type | Aquifer depth range | Include in Supp. Fig. 19 ? | Result *** | Reference |
|---|---|---|---|---|---|---|---|---|
| Southern Hills regional aquifer system, Baton Rouge Area, Louisiana | 30.35 | -91.13 | 2800-foot-Sand | Confined | ~850 m | Yes | Seawater intrusion | Heywood, C.E., Lindaman, M., and Lovelace, J.K., 2019, Simulation of groundwater flow and chloride transport in the "1,500-foot" sand, "2,400-foot" sand, and "2,800-foot" sand of the Baton Rouge area, Louisiana: U.S. Geological Survey Scientific Investigations Report 2019–5102, 49 p., https://doi.org/10.3133/sir20195102. |
| Florida: 80 km of Gulf coastline | 28.75 | -82.52 | Floridan | Unconfined | <65 m | No *** | Seawater intrusion | Mills, L. R., & Ryder, P. D. (1977). Saltwater intrusion in the Floridan aquifer, coastal Citrus and Hernando Counties, Florida, 1975 (No. 77-100). |
| Brunswick, Georgia | 35.16 | -81.49 | Upper Floridan Aquifer | Confined | 180m to 300m | No *** | Saltwater intrusion ** | Cherry, G.S., and Peck, M.F., 2017, Saltwater intrusion in the Floridan aquifer system near downtown Brunswick, Georgia, 1957–2015: U.S. Geological Survey Open-File. Report 2017–2010, 10 p., https://doi.org/10.3133/ofr20171010 |
| Baton Rouge, Louisiana | 30.33 | -91.00 | "1,500-foot" sand, "2,400-foot" sand | Confined | 450m to 750m | Yes | Landward gradient | Heywood, C.E., Lindaman, M., and Lovelace, J.K., 2019, Simulation of groundwater flow and chloride transport in the "1,500-foot" sand, "2,400-foot" sand, and "2,800-foot" sand of the Baton Rouge area, Louisiana: U.S. Geological Survey Scientific Investigations Report 2019–5102, 49 p., https://doi.org/10.3133/sir20195102. |
| West Coast Basin, California | 33.76 | -118.25 | Lakewood, Upper San Pedro and Lower San Pedro Aquifer Systems | Semi-confined to confined | <300 m | Yes | Seawater intrusion | Land, M., Reichard, E. G., Crawford, S. M., Everett, R. R., Newhouse, M. W., & Williams, C. F. (2004). Ground-water quality of coastal aquifer systems in the West Coast Basin, Los Angeles County, California, 1999–2002. US Geol Surv Sci Invest Rep, 5067. |
| Manhattan, New York | 40.72 | -73.99 | Glacial Aquifer | Unconfined | <75 m | Yes | Seawater intrusion | Stumm, F., & Como, M. D. (2017). Delineation of Salt Water Intrusion through Use of Electromagnetic-Induction Logging: A Case Study in Southern Manhattan Island, New York. Water, 9(9), 631. |
| Houston-Galveston Region, Texas | 29.69 | -91.12 | Chicot, Jasper, and Evangeline Aquifers | Unconfined (Chicot, Evangeline); Confined: Jasper | <1,000 m | Yes | Landward Gradient | Braun, C.L., Ramage, J.K., and Shah, S.D., 2019, Status of groundwater-level altitudes and long-term groundwater-level changes in the Chicot, Evangeline, and Jasper aquifers, Houston-Galveston region, Texas, 2019: U.S. Geological Survey Scientific Investigations Report 2019–5089, 18 p., https://doi.org/10.3133/sir20195089. |

* Groundwater fluxes of zero stated in Table 2 of this work for the following watersheds (or coastal segments): Skokomish, Duckabush, Dosewallips, Hamma Hamma, Thorndyke, Tarboo, Dewatto, Big Beef, Anderson (1 and 2), Rendsland, Seabeck, Stavis. Although n=24 numbered shorelines exist in Table 2 only n=23 could be geolocated from Figure 2 (i.e. we do not map shoreline #24 as its location could not be determined)

** Saltwater upconing from Fernandina permeable zone see reference

*** Coastline segment too long for comparison with groundwater well data
